# Enhanced industrial wastewater monitoring: method development for non-target screening of highly polar substances using ZIC-HILIC-HRMS

**DOI:** 10.1007/s00216-024-05635-9

**Published:** 2024-11-16

**Authors:** Reyhaneh Armin, Jan Wachendorf, Markus Weber, Torsten C. Schmidt

**Affiliations:** 1 Faculty of Chemistry, Instrumental Analytical Chemistry, University of Duisburg-Essen, Universitätsstraße 5, 45141 Essen, Germany; 2 Environmental Analysis, Currenta GmbH & Co. OHG, D-51368 Leverkusen, Germany; 3 Chemical Pharmaceutical Analysis - Chromatography-Mass Spectrometry, Currenta GmbH & Co. OHG, D-51368 Leverkusen, Germany; 4 Environmental Analysis, Currenta GmbH & Co. OHG, 41538 Dormagen, Germany

**Keywords:** Highly polar compounds, Industrial wastewater, Zwitterionic hydrophilic interaction liquid chromatography (ZIC-HILIC), Non-target screening (NTS)

## Abstract

Non-target screening (NTS) plays a major role in the monitoring and management of water bodies. While the NTS of moderate to non-polar substances is well-established, the screening of highly polar chemicals remains challenging. In this study, a robust separation method for highly polar substances using zwitterionic hydrophilic interaction liquid chromatography coupled with high-resolution mass spectrometry (ZIC-HILIC-HRMS) was developed. This method was specifically designed for the NTS of industrial wastewater, with the objective of capturing a wide range of polar contaminants in each acquisition run. Method validation included assessing key parameters such as repeatability, reproducibility, linearity, and limit of detection (LOD). For repeatability and reproducibility, the average %RSD of intensity and retention time across all substances in different matrices—solvent, influent, and effluent—remained below 6% and 1%, respectively (*n* = 10). The method demonstrated good linearity (*R*^2^ > 0.99) for 75% of the substances, while LODs varied between 0.1 and 40 µg/L depending on the compound tested. The method was then applied for NTS analysis of untreated wastewater at various locations within a chemical industrial park. Additionally, the overall influent and effluent of an industrial wastewater treatment plant (WWTP) were monitored over a 10-day period. Principal component analysis (PCA) was performed to interpret the data, identifying irregularities in the wastewater content. Moreover, the method demonstrated the WWTP’s ability to achieve an average removal efficiency of approximately 90% for this category of substances in this period, while also detecting their degradation products in the effluent. Finally, the method was successfully integrated into the daily monitoring routine of the WWTP, ensuring continuous surveillance and improved management of wastewater treatment processes.

**Graphical abstract:**

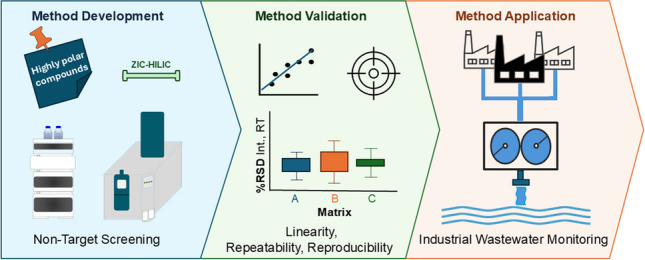

**Supplementary Information:**

The online version contains supplementary material available at 10.1007/s00216-024-05635-9.

## Introduction

The release of anthropogenic chemicals has had a negative impact on the environment and in particular on our aquatic systems [1, 2]. The contamination of water bodies stems primarily from waste disposal practices, agricultural runoff, and industrial discharges [3]. The latter consists of a spectrum of compounds including pharmaceuticals, plant protection products (PPPs), plastics, and their transformation products (TPs). The diverse physicochemical properties of these substances govern their behavior within aquatic environments. Industrial chemicals such as melamine and related triazines, and many pharmaceutical or PPP metabolites and TPs are examples of persistent mobile substances with high solubility in water [4, 5]. A substantial share of the TPs are formed after treatment processes in an industrial wastewater treatment plant (WWTP) [6]. Due to their polar nature and their increased mobility in water bodies, their removal poses challenges, even in the presence of advanced treatment procedures [7, 8]. Unlike non-polar compounds, they are not effectively removed through sorption processes, leading to their ubiquitous occurrence in aquatic systems and raising concerns about water quality. Because of the difficulties in treating and removing these substances, there is a risk they may reach our drinking water, potentially causing health hazards [6]. Therefore, the close monitoring of polar persistent compounds, especially at their release source, is of great importance.

Thanks to advances in analytical chemistry, a vast number of emerging anthropogenic substances have been identified and are currently being monitored in various water matrices, including surface water, groundwater, and wastewater. Targeted analysis via liquid chromatography coupled to tandem mass spectrometry (LC-MS/MS) allows the quantification of known compounds at very low concentration ranges (ng/L to μg/L) [9–11]. In recent years, suspect and non-target screening (NTS) techniques via high-resolution mass spectrometry coupled to LC (LC-HRMS) have also been increasingly utilized in environmental analysis. These methods allow for the comprehensive analysis of sample composition without prior knowledge of the constituents present. NTS is a powerful tool capable of detecting unknown compounds [12]. It has proven particularly effective in the identification of initially unknown metabolites and TPs [13–15]. The majority of the established NTS techniques employ reversed-phase liquid chromatography (RPLC) with a C18 column, which is an extremely robust separation method for organic non-polar to moderately polar compounds. In addition to the traditional C18, innovative RP stationary phases featuring polar end-capping have been developed. Their aim is to enhance the separation of substances with higher polarity, thereby enabling the analysis of a broader spectrum of compound classes [6, 16]. Nevertheless, when injected on an RP, highly polar compounds tend to elute together in the void volume, as there is minimal to no interaction between these and the non-polar stationary phase. Therefore, RPLC has proven ineffective in retaining highly polar analytes [6].

Numerous techniques have been developed for the separation of highly polar compounds. However, analyzing such compounds within an aqueous matrix presents distinct challenges in contrast to non-polar substance analysis, including ion suppression and competition with water for the stationary phase [17–19]. Analytical methods suggested for the analysis of polar substances include supercritical fluid chromatography (SFC), ion-chromatography (IC), and mixed mode liquid chromatography (MMLC) [20–24]. Most commonly used though is hydrophilic interaction liquid chromatography (HILIC) [25]. A few drawbacks, such as long equilibration times, excessive column bleeding, broad chromatographic peaks, and issues with robustness, are associated with HILIC [12, 17, 19, 26]. Despite these issues, HILIC remains one of the most promising analytical techniques for this purpose. Zwitterionic HILIC columns (ZIC-HILIC), containing positively and negatively charged functional groups, are an extension to traditional HILIC and offer a better separation of ionic compounds based on ion-exchange mechanisms [18, 27].

Over the recent years, HILIC has often been applied in targeted analysis to quantify polar substances in water [23, 28]. A few studies have combined HILIC and NTS to broaden the analytical window [15, 29, 30]. For example, Kolkman et al. [31] utilized HILIC-NTS to assess the presence of polar contaminants in Dutch and Flemish drinking water. In another example, Minkus et al. [29] developed a two-dimensional LC method using RPLC-ZIC-HILIC-HRMS to perform NTS of compounds spanning non-polar, highly polar, and ionic categories within one measurement. Kochale et al. proposed a column switching method and reported an optimized approach towards the combination of HILIC, RPLC, and NTS [32]. Apart from ZIC-HILIC columns, MMLC has also delivered promising results when coupled to NTS, offering the separation of ionic substances while simultaneously retaining non-polar compounds [33, 34].

While advances in analytical chemistry have opened new doors to monitor persistent mobile polar substances in aquatic systems, preventing their entrance into the environment is the best solution to reduce their presence. Nonetheless, most current studies focus on surface waters post-contamination, rather than on preventive measures. A handful of studies have implemented NTS for contamination source tracing aiming to reduce response times and enhance the effectiveness of monitoring programs [35–37]. Conducting routine NTS investigations directly at an emission source, such as an industrial WWTP, enables faster response times and mitigates the release of polar contaminants. However, a robust separation technique for this class of compounds is still lacking. By developing a method tailored for industrial wastewater and integrating it with the routine NTS, the release of known and unknown highly polar persistent compounds can be prevented. This study aims to develop and optimize such a technique and utilize it to monitor the influent and effluent of an industrial WWTP.

## Materials and methods

### Chemicals and reagents

Twenty reference substances were used for method development, a list of which can be found in Table S1 of the supplementary material. The nature of these could be divided into four groups: acidic, basic, neutral, and amphoteric. All substances had a purity of ≥ 95%, with log D values ranging from − 7 to 2 at pH 7.4. The exact log D values are presented in Fig. S1. Stock solutions with a concentration of 1000 mg/L were prepared in Milli-Q ultra-pure water from Merck (Darmstadt, Germany). These were kept at 3 °C. Few substances were not completely soluble in water alone, so a small percentage of LC/MS grade methanol from Fisher Scientific (Geel, Belgium) was used. Along with the methanol, LC/MS grade acetonitrile from CHROMASOLV™ Honeywell (Seelze, Germany) and Milli-Q ultra-pure water were used as eluents throughout this study. The buffer solution used was the LiChropur™ ammonium acetate ≥ 99% LC/MS grade from Sigma-Aldrich (Darmstadt, Germany). Acetic acid reagent grade ≥ 99% from Honeywell (Seelze, Germany) and formic acid ≥ 99% HiPerSolv Chromanorm (VWR Chemicals, UK) were used to adjust the pH value of the buffer solution.

### Preparation of quality control samples

Twenty microliters from each of the stock solutions was aliquoted into Milli-Q water, resulting in the formation of a 20-mL mix solution containing all reference substances at a concentration of 1 mg/L. Quality control (QC) samples were prepared by diluting this mix solution by a factor of 10 with acetonitrile (ACN), to achieve a concentration of 100 μg/L. The QC samples were used for the development of the separation method and feature extraction parameter optimization. QC samples were also measured at the beginning and at the end of each sequence during the measurement of the wastewater samples to monitor instrumental drifts.

### Sampling sites and sample preparation

**Fig. 1:**
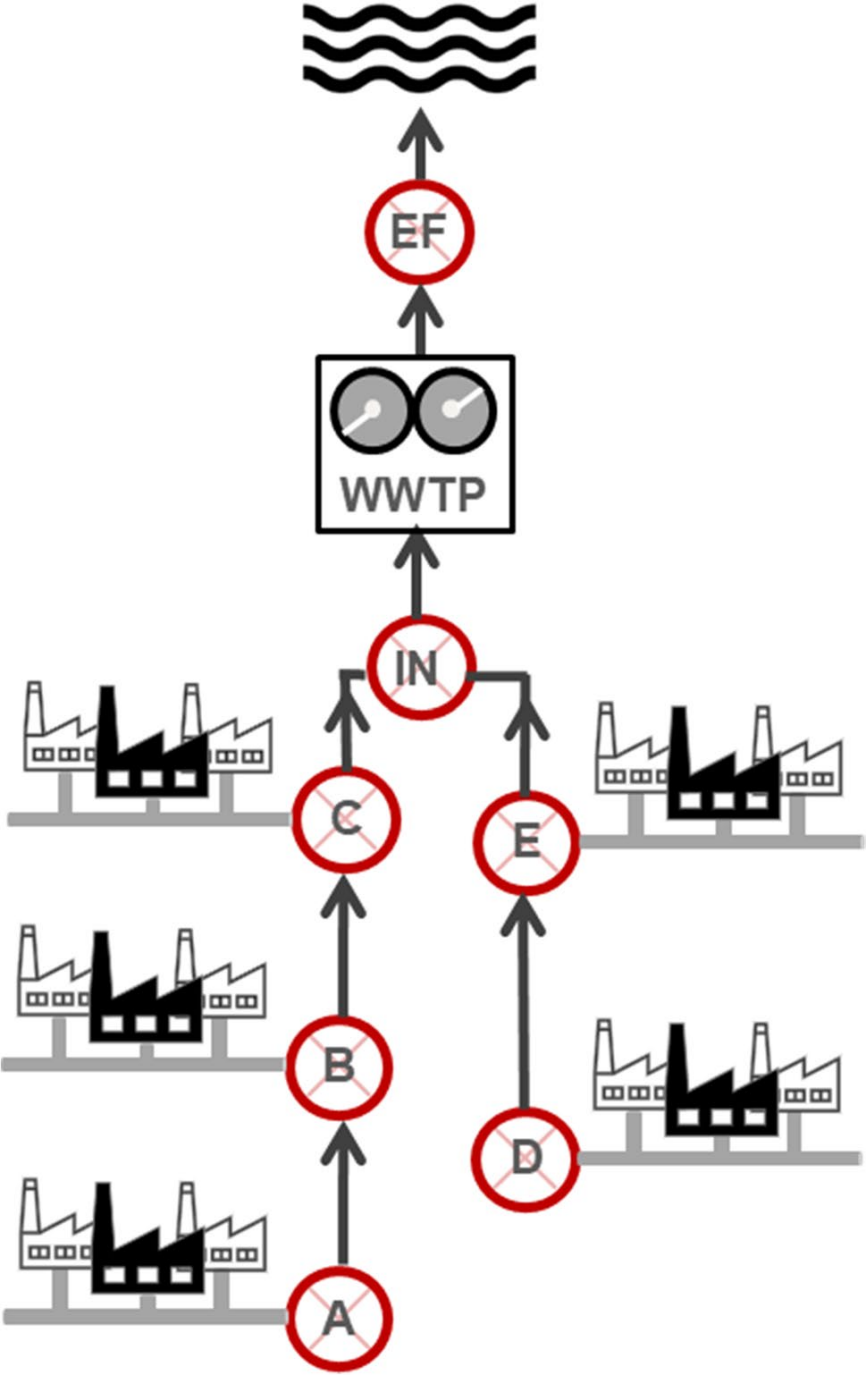
Schematic representation of the wastewater system within a chemical industrial park. The wastewater system consists of five sections. Wastewater from each plant flows to the junctions (A–E), where sampling stations are available. Sampling stations are also placed at the influent and the effluent (IN, EF). From each sampling site, 24-h composite samples were taken, which are indicated with a red mark. This figure serves an exemplary purpose and is not to scale. The basic overview of the treatment process involves grit removal, neutralization, sedimentation, 1st biological treatment followed by denitrification and separation of activated sludge, and 2nd biological treatment. Finally, the activated sludge is again separated, and the treated water is discharged

The sampling was conducted in a chemical industrial park, containing roughly 50 chemical plants and an industrial WWTP. As depicted in Fig. 1, the wastewater system is divided into five sections. The wastewater of multiple plants flows to the junctions (A through E), where sampling stations are available. Sampling stations are also placed at the WWTP influent (IN) and effluent (EF). Due to proprietary reasons, the content of the wastewater has to remain confidential throughout this work.

Twenty-four-hour composite samples over 10 consecutive days from the influent (IN) and effluent (EF) were employed for monitoring purposes. To trace the substances within the industrial park back to each segment, one 24-h composite sample of each junction (A–E) was also obtained during the same time frame as the influent/effluent sampling. Samples were measured within a 2-day interval from sampling date. If measurement was not possible on the same day as sampling, the samples were kept at 1–5 °C.

The metadata indicated that the untreated samples contained high concentrations of chemicals; thus, in contrast to well-established sample preparation for clean-up and enrichment by solid-phase extraction, we could dilute the samples prior to injection. Samples from the influent and the junctions were diluted with ACN by a factor of 10 prior to measurements. Typically, due to the lower contamination load in the effluent, these samples are measured undiluted. However, injection of effluent samples into a HILIC column presented challenges due to the highly polar nature of the matrix. Undiluted injection led to analyte partitioning into the stationary phase, causing chromatographic peak distortion. Therefore, three dilution factors using ACN were examined: 1:2, 1:5, and 1:10. The dilution process led to the formation of significant quantities of precipitate, caused by the reduced solubility of salts present in the sample. Thus, a filtration step was added to the sample preparation process. Although the dilution factors 1:2 and 1:5 did cause a minor improvement in the peak shape, the results were still not satisfactory. It was therefore decided to also dilute the effluent samples by a factor of 10 with ACN. The dilution process unfortunately results in a sensitivity loss of the analytical system.

Paracetamol, chlormequat chloride, and metformin (with log D values 0.90, − 3.31, and − 5.62, respectively) were added to the junctions and influent/effluent samples in specific quantities to achieve a final concentration of 50 μg/L in the sample matrix. This was carried out for the non-target studies. Due to difficulties obtaining deuterated substances, these three compounds were used as internal standards. All three were detected and used in positive mode, with paracetamol being the only standard for measurements in negative mode. The absence of these substances in the wastewater matrix was established based on prior metadata analysis. These compounds were used to account for matrix effects arising due to the changes in the wastewater matrix over the 10-day period. Parameters such as intensity, retention time (RT), full width half maximum, (FWHM), asymmetry factor, and mass error were assessed after each non-target sequence.

### Instrumentation and acquisition

Overall, three columns were used. (1) a C18 column and precolumn with an already established method used for comparison [38]. This was the Trident cartridge (10 × 2.1 mm) and filter (2 mm, 0.5 µm) precolumn and an Ultra Aqueous C18 (100 mm × 2.1 mm, 3.0 µm particle size) column, both from Restek GmbH (Bad Homburg v. d. Hohe, Germany). (2) A mixed mode column from Thermo Scientific, namely the Acclaim Trinity P1 (2.1 μm, 100 × 3 mm) (Waltham, USA). (3) A zwitterionic hydrophilic interaction (ZIC-HILIC) column, specifically the SeQuant ZIC-HILIC from Merck (3.5 μm, 200 Å, 150 × 2.1 mm). A pre-column SeQuant ZIC-HILIC Guard 5 μm, 200 Å, 20 × 2.1 mm was also used in this setup (Darmstadt, Germany).

For the analysis of the wastewater samples, a method employing the ZIC-HILIC column was developed and optimized. The steps to the development and finalization and validation of the method are discussed in detail throughout this manuscript. In the final method (method 3, Table S2), eluent A consisted of an aqueous 20 mM ammonium acetate solution, while eluent B comprised a 95/5 ACN/H_2_O mixture also with 20 mM ammonium acetate. The pH of the eluents was 6.8. The duration of the method was 30 min, where the last 10 min was reserved for column equilibration. 0.5 μL of the sample was injected into the HPLC system. The flow rate was set at 0.3 mL/min, with a column oven temperature maintained at 35 °C. The gradient program is described in Fig. S5 (gradient 1) of the supplementary material.

In the case of MMLC, the method proposed by Montes et al. [22] was employed. The acquisition parameters for RPLC are described by Purschke et al. [38]. These methods are discussed in more detail in Sect. 4 of the ESM.

The HPLC system was a 1260 Agilent Infinity (Agilent Technologies, Waldbronn, Germany), consisting of an autosampler, binary pump, DAD module, and column oven. An X500R QTOF high-resolution mass spectrometer with a Turbo V ESI source (SCIEX GmbH, Darmstadt, Germany) was used to acquire HRMS data. More information on HRMS settings is provided in Table S3. All wastewater samples were measured in triplicates. After every fifth measurement within the sequence, a blank (ACN) was measured. Internal mass calibration was carried out throughout the sequence. Data acquisition was carried out with the SCIEX OS software (version 1.7, SCIEX GmbH, Darmstadt, Germany).

### Univariate method development and optimization

To establish an optimum separation method for our purpose, the influence of several parameters such as the stationary phase, mobile phase, buffer concentration, pH value of the mobile phase, gradients, column oven temperature, and flow rate was investigated. For this purpose, the QC, one influent and one effluent samples were used, and the influence of each parameter on the separation of the reference standards was examined. The method development was carried out in wastewater, as studies have shown how in case of HILIC columns, the matrix can have an extreme influence via ion suppression [19]. The influent and effluent samples were prepared by adding 100 μL of the mix stock solution and 100 μL of the sample (influent or effluent) to 800 μL of ACN, followed by measurement.

### Repeatability and reproducibility

To examine the repeatability (intraday precision) of the final method, a QC sample was measured 10 times in one sequence. The percentage relative standard deviation (% RSD) for the intensity and the retention time of the reference substances were calculated. Additionally, the method’s repeatability was verified in the influent and effluent matrices. For this purpose, the samples were prepared as mentioned in the “Univariate method development and optimization” section.

The reproducibility of the method (interday precision) was also examined. The same procedure as above was carried out, where 10 QC samples were measured over 2 weeks (*n* = 10).

### Linearity and limits of detection

The mix solution was diluted with ACN to prepare samples with concentrations between 1 and 100 μg/L. These were measured and calibration curves were constructed to determine the linearity of the analytical method. Limits of detection were defined as the concentrations corresponding to a signal-to-noise ratio of 3, using mixed solutions that were further diluted with acetonitrile (ACN) to final concentrations of 500 ng/L and 100 ng/L.

### Target and non-target data analysis

**Fig. 2:**
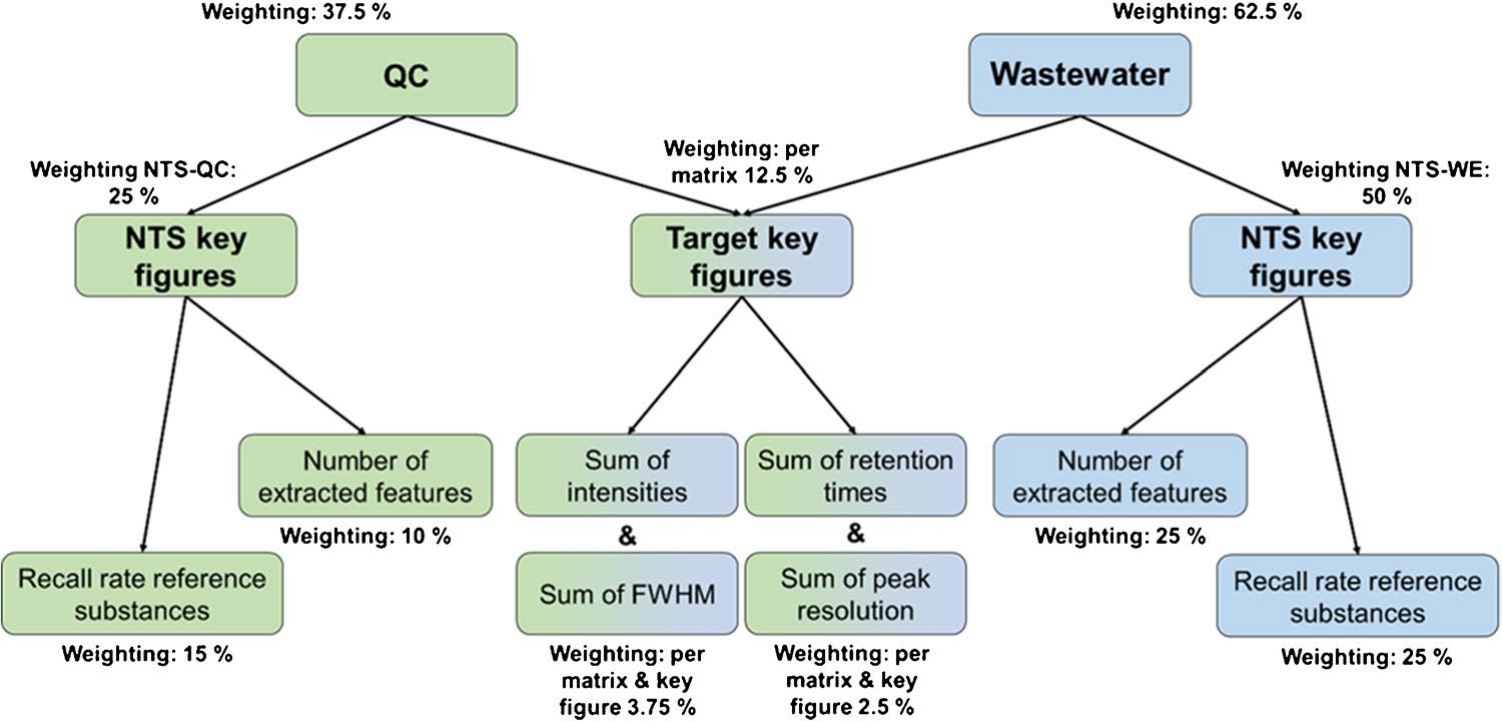
Scheme describing the implemented scoring approach. This assisted in the ZIC-HILIC method performance comparison of the tested methods. The approach was carried out after each method development iteration and measurement of QC and spiked wastewater samples

The mentioned SCIEX OS software was used for target analysis and identification of reference substances. Following each method optimization iteration, SCIEX OS was employed to retrieve data on detected mass-to-charge ratio (*m*/*z*), mass error, RT, intensity, and FWHM. These parameters were critical metrics for assessing the method and improving subsequent iterations and were used in the scoring system (Fig. 2, and Sect. 2, supplementary material).

Non-target analysis or feature extraction was carried out with MZmine3 (3.9.0) [39, 40]. Similar to targeted analysis, a feature extraction workflow was performed on every measurement after each step of method development or optimization. It was crucial that the feature extraction software also showed a satisfactory performance for the tested method. For example, it was observed that methods yielding broad peaks or those with even minimal splitting caused difficulties for MZmine3 to create an accurate chromatogram. This occurred despite feature extraction adjustments. The feature extraction parameters for the finalized separation method are listed in Table S5 of the supplementary material.

To perform feature extraction, firstly, the .wiff2 data generated by the SCIEX instrument were transformed to .mzml format with MSConvert within Proteowizard (3.0) [41]. The .mzml data were imported to MZmine3. The feature extraction workflow consisted of the following steps: (1) mass extraction, (2) chromatogram building, (3) chromatogram smoothing, (4) chromatogram deconvolution, (5) isotope and adduct removal, (6) alignment of the triplicates and blanks, (7) removal of features not present in all triplicates, and finally (8) background removal. The final feature lists were exported in a .csv format. The 10-day influent data were aligned to create one list including all features and their respective intensities measured over this time period. The same procedure was carried out with the effluent samples. To examine the removal efficiency of the WWTP by comparing the composition of the influent and effluent over the 10-day time period, these data were also aligned to create one comprehensive influent-effluent data matrix.

### Evaluation and selection of the most suitable method

Given that this method was intended for non-target analyses, several additional factors needed consideration to select the most suitable one for our objectives. Following each measurement with a respective tested method, both target and non-target workflows were executed with QC and spiked wastewater samples, and a “scoring” approach was undertaken. RT, intensity, peak resolution, and FWHM from the target analysis, and recall rates obtained from the non-target workflow were the “key figures” contributing to the scoring system, which is depicted in Fig. 2. Of course, some of the essential non-target workflow parameters were optimized for each method (see “Target and non-target data analysis”). Each key performance indicator was assigned points based on its quality and relevance. To achieve a compromise and balanced selection, a weighting system was applied to the scores, with higher weights assigned to the method’s performance with wastewater samples since this matrix is critical to the study. The QC, however, remained important for evaluating overall performance in a neutral environment, providing a broader perspective, and ensuring that the method was not limited to this specific wastewater matrix, as the composition of wastewater can change over time. Attention was paid to the method’s influence on key figures of the targets, such as retention time and intensity. Nevertheless, the NTS workflow was given an even greater emphasis, particularly with respect to the compatibility of feature extraction software with the generated data. Initial experiments were conducted in positive mode, and the three best-performing methods were selected based on the scoring approach. Measurements were repeated in both modes with the three final methods. The scoring procedure was subsequently applied for both positive and negative data, with slight modifications to the scoring criteria, as shown in Fig. S6 (section 2) of the supplementary material, in order to select the final method.

### Application of principal component analysis (PCA)

The .csv influent and effluent data matrices exported from MZmine3 were imported to the MATLAB environment (MathWorks Inc., version 9.13, 2022b). Logarithmic transformation, autoscaling, and normalization were performed as preprocessing steps. Afterwards, both datasets were subjected to PCA separately to identify any major events in the chemical composition of the samples.

### Calculation of the removal efficiency

The WWTP’s removal efficiency was determined by examining the intensities of the features found in the influent and effluent samples. The average intensities of the features over the 10-day period were first calculated, for both influent and effluent separately. The removal efficiency for each feature was then calculated with the following equation:

$$\%Removal\;Efficiency=\frac{I_{inf,avg}-I_{eff,avg}}{I_{inf,avg}}\times100$$

## Results and discussion

### Method development and optimization

To develop and optimize a method for the separation of polar substances, different factors such as the stationary phase, eluent composition including different buffer concentrations and their pH, as well as gradients were tested. Method development was initially carried out using QC samples, as well as influent and effluent samples spiked with standards. The influence of each parameter was then evaluated and quantified using a scoring system. Considering the intended coupling of this separation method with NTS, we aimed to develop a suitable approach for multicomponent analysis, hence, to detect and separate as many compounds as possible. The investigations were initiated using ACN and ultra-pure water as the mobile phase system, following the recommendation of the column manufacturer and supported by numerous studies demonstrating the successful separation of polar compounds via LC-MS [18, 29, 31, 33].

#### Stationary phase

Two stationary phases for the separation of the polar substances were tested: the Trinity Acclaim mixed mode column from Thermo Fisher, and the SeQuant ZIC-HILIC from Merck. According to recent studies, both columns have shown a great potential in separating ionic and polar compounds [20, 33]. These columns, however, are unfortunately prone to high column bleeds [17]. Prior to method optimization, a test run with a QC sample was conducted with both of these columns employing methods proposed by Montes et al. [33] (for MMLC) and Boulard et al. [18] (for ZIC-HILIC) to observe their initial performance.

##### Mixed mode column

The column was equilibrated as recommended by the manufacturer. Despite a relatively long equilibration period, the first measurements revealed extremely high amounts of column bleeding. The column bleeds had a negative influence on the performance of the HRMS and caused issues with the ion source. Due to ion suppression, most of the reference standards were detected with extremely low intensities. This also caused low robustness in the measured intensities. The total ion current (TIC) of one measurement is shown in the supplementary material, Fig. S2. It was decided to eliminate this column from further studies to avoid problems with the HRMS.

##### ZIC-HILIC column

The same procedure as above was carried out for the ZIC-HILIC column. High column bleeds were at first observed with this column as well; however, after sufficient equilibration, they were reduced to an acceptable level. Further optimization experiments were only performed on the ZIC-HILIC column.

#### pH of the buffer system

Buffer pH values of 7.5, 6.0, and 4.5, along with the pH value of the non-adjusted ammonium acetate solution (6.8), were examined in positive mode. During this investigation, an isocratic method with a mobile phase 90/10% ACN/H_2_O + 20 mM buffer concentration was used.

The achieved retention times remained consistent across different pH values. At lower pH levels, an increase in the FWHM was observed. Peak intensity was highest for most substances at pH 6.8 and 6.0 but notably decreased at both higher and lower pH values, resulting in decreased sensitivity. For instance, melamine exhibited an intensity of 5.3E + 04 at pH 6.8, followed by 3.0E + 04 at pH 6.0, while at pH values 4.5 and 7.5, the method yielded intensities of 1.8E + 04 and 9.0E + 03, respectively. This was not the case for chlormequat and metformin, which showed slightly higher intensities at pH 6.0, representing the highest values recorded for both compounds. The pH 6.8 yielded in narrower chromatographic peaks, with lower FWHM values, particularly in the case of later eluting compounds, such as gabapentin. As a result, the pH value of 6.8 (the initial pH value of the ammonium acetate buffer) was selected for further method development. This value yielded the best results with the NTS workflow. For certain compounds, such as aspartame, the observed sensitivity at pH 7.5 and 4.5 was significantly reduced, leading the feature extraction software to classify these signals as background noise. Additionally, at these pH values, the software was unable to extract gabapentin, due to the extreme broadness of the XIC. Additional information regarding the impact of pH values on intensities, RT, and FWHM are provided in Fig. S3, depicting the XICs of selected compounds at the investigated pH values.

#### Concentration of the ammonium acetate buffer

For the examination of the buffer ionic strength, buffer concentrations ranging from 5 to 20 mM were evaluated. These were tested under isocratic conditions in positive mode. Unlike pH values, buffer concentration significantly affected chromatographic separation for some substances. The retention times of early eluting substances were not strongly influenced by this parameter. On the other hand, later-eluting substances demonstrated a significant increase in retention time with decreasing buffer concentration. This was particularly the case for strongly basic or cationic compounds like chlormequat and metformin. For the latter, retention times of 18.4 min at 5 mM, 12.7 min at 10 mM, and 9.2 min at 20 mM were observed. However, with a decrease in the buffer concentration, an extreme increase in the FWHM and decrease in the intensities were observed, which caused challenges in the NTS workflow. Looking closely at metformin again, a FWHM of 0.6, 0.4, and 0.25 was calculated for 5, 10, and 20 mM buffer, respectively. In conclusion, a buffer strength of 20 mM gave the most promising results. Extracted ion chromatograms (XIC) of exemplary substances measured at 5, 10, and 20 mM buffer concentration are illustrated in the supplementary material, Fig. S4.

#### Final method

Following the establishment of the mobile phase composition, 18 further methods were tested, where the oven temperature, the flow rate, and gradients were optimized. A detailed table including these parameters can be found in the supplementary material (Fig. S5 and Table S2).

Of the 18 tested methods, numbers 2, 3, and 14 delivered promising results based on the scoring system mentioned in the “Evaluation and selection of the most suitable method” section. While all three methods exhibited satisfactory performance, it was observed that method 3 demonstrated a slightly better separation of the cationic/basic substances in the complex influent and effluent matrices. This method also showed the highest compatibility with the MZmine3 (NTS) workflow, exhibiting the highest recall rates across quality control (QC), influent, and effluent samples when processed through the software. Consequently, method 3 was selected as the preferred method for conducting further experiments. It is noteworthy that the reference standard glyphosate was not detected with either method. Additionally, despite optimization efforts, the separation of the substances 1,3 di-o-tolylguanidine and 3-amino-1,2,4 triazole remained a challenge. Substances acquired in negative mode, such as cyanuric acid and 5-fluoroacil, showed relatively lower intensities, due to the lower sensitivity of the HRMS instrument in this mode. The final method is also mentioned in the “Instrumentation and acquisition” section of “Materials and methods.”

**Fig. 3:**
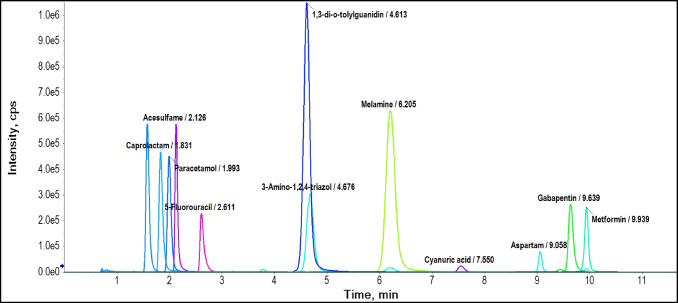
Selected extracted ion chromatograms (XICs) of reference standards in QC samples acquired with the final method (method 3) in ESI (+) and (−) modes

XICs of the selected substances obtained with method 3 in positive and negative modes are visualized in Fig. 3. As expected, opposed to the classic C18 method, compounds with a higher log D value such as cardiol and caprolactam (RT: 1.5 and 1.9 min, respectively) were eluted earlier than melamine and metformin (RT: 7.2 and 10.3 min, respectively), two examples of compounds with low log D values. In general, with the established method basic and amphoteric substances showed the highest retention (minutes 6–10), whereas acidic substances were eluted earlier (minutes 1.5–3).

### Repeatability and reproducibility

Prior to applying the developed method to real data and WWTP monitoring, it was crucial to assess the repeatability and reproducibility of the method in both positive and negative modes. This was particularly important since HILIC separation methods are usually associated with a lack of robustness, especially over longer periods of time [42]. Given that this method should be applied for NTS workflows, it was of great interest to focus on the robustness of the RT values which play a major role in the alignment of measurements. Intensity values are another important factor, as these values are used for trend analysis or quantitative screening analyses. Given the dependency of the NTS workflow on intensity values rather than peak areas, %RSD of the peak areas was not considered.

**Fig. 4:**
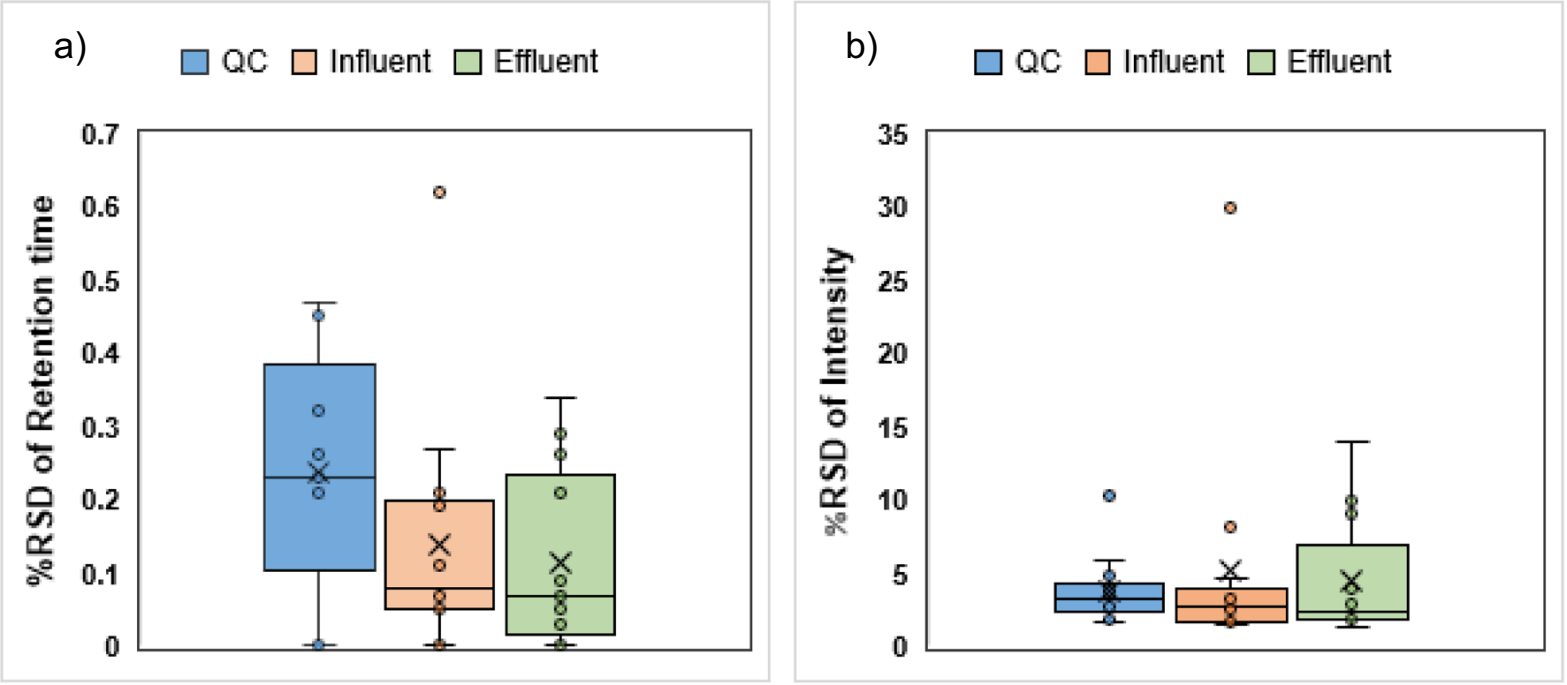
Boxplots representing the range of calculated % RSD for **a** RT and **b** intensity of the compounds in positive and negative modes (*n* = 10) to determine the repeatability of the separation method (method number 3). The repeatability experiments were carried out in solvent (QC) and two wastewater matrices

Repeatability or intraday precision of the reference substances was carried out in solvent and two further matrices, namely QC, influent and effluent (*n* = 10). The % RSD of the substances’ intensities and RTs were calculated and are presented in Fig. 4.

In general, the average % RSD of the standard compounds’ RT was < 0.3 in the solvent and the wastewater matrices. This low value is satisfying for a HILIC separation method. As Fig. 4a and b suggest, the highest %RSD was interestingly observed in the QC. A closer look at the RT %RSD of each substance in Fig. S7(B), acephate showed high retention time shifts in the influent matrix, whereas caprolactam showed near zero %RSD in all samples.

Although the %RSD of the intensities were substantially higher compared to the RT, they still had an average value of < 5 in wastewater matrices and the solvent, which is a satisfactory outcome. Similar to the RT study, acephate was detected with significant shifts in its intensity across all three samples (Fig. S7(A)).

Reproducibility or interday precision was carried out only with the QC. The experiment was conducted exclusively in solvent due to the uncertainty surrounding the consistency of influent and effluent matrices over the course of 10 days. Daily samples have fluctuating matrices, and the chemicals within these samples are prone to degradation over time. This inconsistency and instability would interfere with the experiments and introduce uncertainty to the results.

**Fig. 5:**
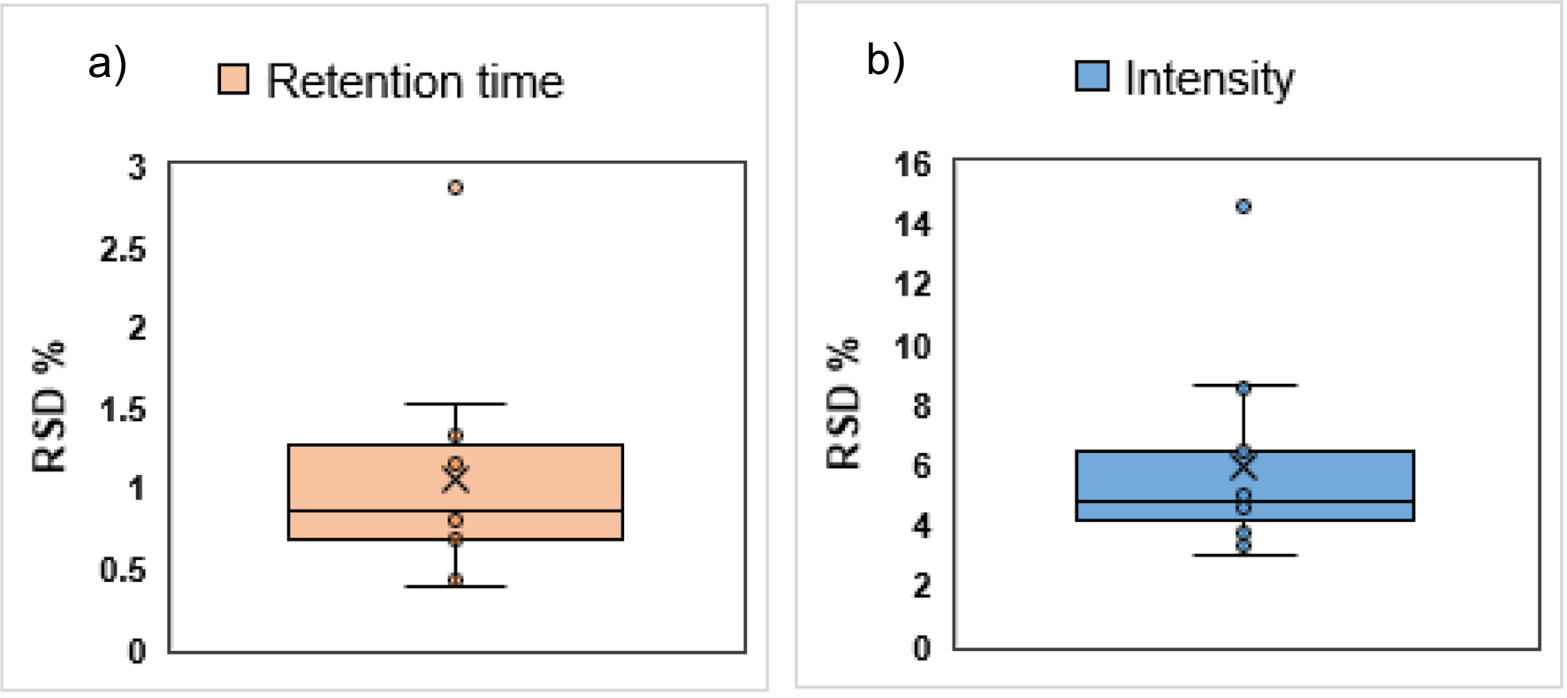
**a** Boxplots representing the calculated % RSD of **a** RT (orange) and **b** intensity (blue) to determine the reproducibility of the method over 10 days in QC samples

The QC sample was measured once a day over a period of 10 days. The RT and intensity %RSD of the substances are presented in Fig. 5. There were no major shifts in the RTs of the substances, with the average of %RSD at 1. However, the intensity values exhibited less stability, with an average %RSD of 6. The intensities of specific substances, such as acephate, thiourea, and aspartame were responsible for this discrepancy (Fig. S8). It is important to note that the performance of the HRMS system also plays a role in the obtained results. To ensure this matter, multiple internal calibration of the HRMS within each sequence was carried out.

In conclusion, the developed method showed acceptable repeatability and reproducibility in the case of most substances. For some compounds such as acephate and thiourea however, high %RSDs were observed.

### Linearity and limits of detection

Considering that this method is to be applied for trend analyses and potential quantitative screening, it was crucial to evaluate the linearity and LOD of the method. This study aimed to provide a general understanding of the method’s detectability LOD across a range of different compounds, helping us evaluate its overall performance. Although future analyses will be performed on wastewater samples, it was decided to conduct the linearity and LOD experiments using QC samples. This was due to the fact that the influent wastewater matrix may already contain the target substances, potentially causing uncertainties or interferences in the investigation*.* In general, the established method provided good linearities and low LODs for most of the substances, the results of which are presented in Table 1.

**Table 1:** The linearity presented through the correlation coefficient (*R*^2^) of the calibration curve and LOD of the reference compounds were investigated with the established method. Compounds measured in negative mode are indicated with (−). The LODs of 1,3-di-o-guanidin and saccharin (−) are estimated at ≤ 0.1 μg/L based on the achieved S/N

Compound	LOD (μg/L)	Correlation coefficient (*R*^2^)	Linearity range (μg/L)
*1,3-Di-o-guanidine*	< 0.1	0.986	1–60
*3-Amino-1,2,4-triazole*	1	0.997	1–100
*MTTA*	10	0.996	10–100
*5-Fluorouracil (* −*)*	5	0.998	10–100
*Acephate*	40	0.999	40–100
*Acesulfam-K (* −*)*	1	0.995	1–100
*Aspartame*	5	0.998	10–100
*Caprolactam*	0.5	0.999	1–100
*Cardiol*	0.5	0.998	1–100
*Chlormequat*	0.5	0.996	1–100
*Cyanuric acid (* −*)*	40	0.916	40–100
*Gabapentin*	1	0.996	1–100
*Melamine*	0.1	0.989	1–100
*Metformin*	1	0.996	1–100
*Paracetamol*	0.5	0.988	1–100
*Perfluorooctanoic acid (* −*)*	10	0.992	10–100
*Picrylsulfonic acid (* −*)*	1	0.69	1–60
*Saccharin (* −*)*	< 0.1	0.995	1–60
*Thiourea*	10	0.995	10–100

The method was further assessed by calculating the LOD at a S/N of 3, with the lowest examined concentration at 0.1 μg/L. Overall, twelve compounds presented an LOD of ≤ 1 μg/L. Specifically, at a concentration of 0.1 μg/L, 1,3-di-o-guanidine and saccharin both presented intensities with S/N ratios ≫ 3. In contrast, the method achieved a high LOD of 40 µg/L for acephate and cyanuric acid.

Furthermore, an 8-point calibration curve was constructed for each substance covering a concentration range of 1 to 100 μg/L. However, for substances with higher LODs, calibration curves were constructed using fewer points. Analysis of the correlation coefficients (*R*^2^) derived from these calibration curves revealed satisfactory linearity for all substances, with coefficients around 0.99. An exception was observed for cyanuric acid and picrylsulfonic acid where the *R*^2^ value fell to 0.91 and 0.69, respectively. The linearity range varied between substances. While some showed linearity covering the full tested concentration range and possibly even beyond, others had a narrower linear range. This study demonstrates that while a multicomponent method for polar compounds is feasible, it faces challenges with some substances due to differences in generated signal intensities across the compounds.

### NTS of polar substances in industrial wastewater

#### Creation of a chemical fingerprint for the junctions

**Fig. 6:**
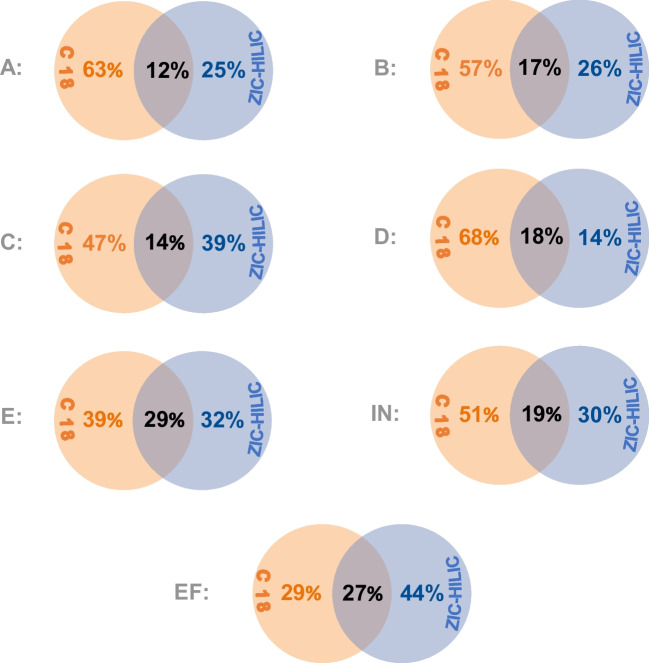
NTS was employed to measure one single 24-h composite sample from each of the five junctions A–E, the influent (IN) and the effluent (EF) with both the C18 (orange) and the established ZIC-HILIC (blue) method. The Venn diagrams display the percentage of features detected only with the C18, the percentage of features detected only with the ZIC-HILIC, and percentage detected with both columns. These outcomes are calculated based on only the exact masses with a deviation of ± 3 ppm

Firstly, one 24-h sample from each junction, one influent, and one effluent sample were measured with the routine RPLC and with the established ZIC-HILIC method. NTS was conducted on both datasets, and the feature lists were compared by matching exact masses within a deviation of ± 3 ppm (Fig. 6). Between 12 and 29% of features were separated successfully with both columns. This outcome was also well expected, as the Restek Aqueous C18 column has polar functional groups offering a better retention of some polar components. Nevertheless, in all five junctions, some features were exclusively detected using the ZIC-HILIC column, highlighting the need to incorporate a method targeting polar compounds into the routine NTS monitoring of industrial wastewater.

The effluent sample exhibited the highest percentage of features detected exclusively with the ZIC-HILIC method, accounting for 44%. In this sample, 27% of the overall features were detected by both methods. This outcome was anticipated, considering that the effluent matrix contains persistent polar substances that may be generated during the treatment process.

With the ZIC-HILIC column, 455, 480, 906, 818, and 1137 features were detected for junctions A through E, respectively. An in-house database was created, consisting of a library for each junction, listing the *m*/*z*, RT, and an ID for every feature indicating the name of the junction where the feature was detected. Therefore, a “chemical fingerprint” was created for A through E, aiming to track the features in the influent and effluent back to their point of origin.

#### Exploration of the chemical space

The influent and effluent of an industrial WWTP were monitored over a 10-day time frame. Consecutive 24-h composite samples were measured with the ZIC-HILIC method. The NTS workflow was carried out and features were extracted. As an additional filtering step, all features with a retention time of < 2 min were removed from the feature lists, to focus on substances with higher polarity. This reduced the number of features in the influent by 63% and in the effluent by 40%. It is important to note that the quality of the generated data was assessed prior to data analysis, with the detailed results presented in Sect. 6 of the supplementary material.

**Fig. 7:**
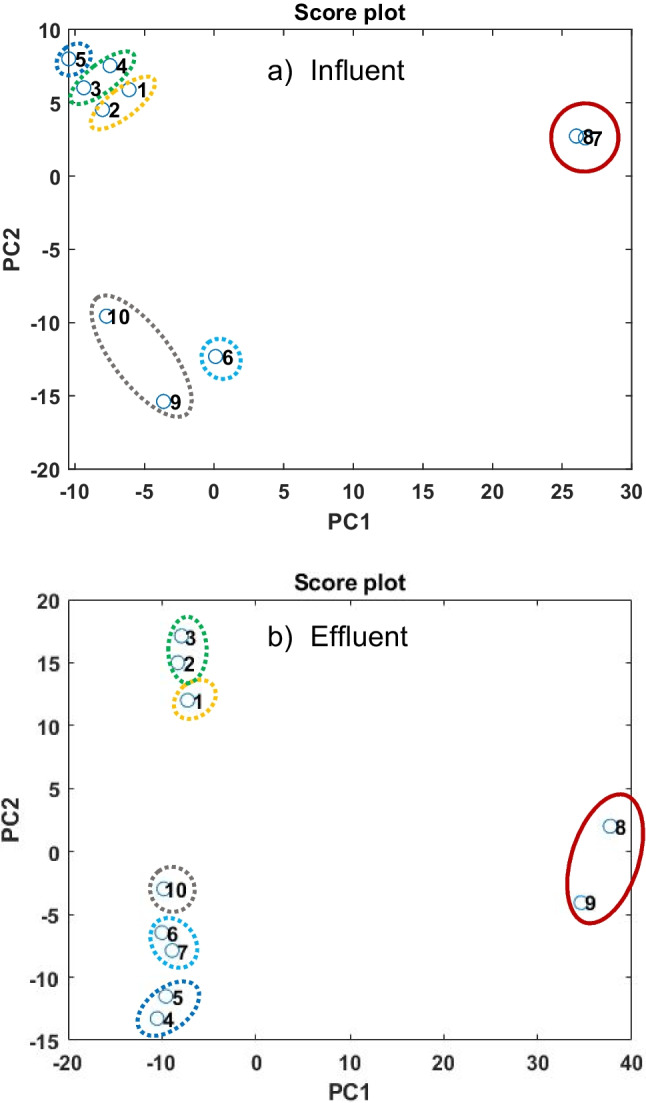
Ten consecutive 24-h composite influent and effluent samples were measured with the established ZIC-HILIC NTS approach. Features were extracted, preprocessed, and subjected to PCA. PCA score plots of **a** influent and **b** effluent samples over 10 days are presented

The influent and effluent data matrices were subjected to PCA individually to visualize the data and to identify any events or patterns within this time frame. The score plots are visualized in Fig. 7a and b.

The score plot of the influent suggests that the features in these samples remained mostly consistent in composition and behavior from days 1 through 5, indicating stability over this period. Additionally, a grouping tendency or a “pattern” was observed every 2 days suggesting a potential temporal trend or correlation. For example, data points corresponding to days 1 and 2 demonstrate proximity to each other, as do data points representing days 3 and 4. The score plot also indicates that starting at the 6th day, there were changes in the composition of the influent. These changes were further intensified on days 7 and 8. However, towards the end of the investigation period, particularly on days 9 and 10, the composition began to resemble that of day 6. Upon closer examination of the dataset, it was apparent that from day 6 onwards, a cluster of new features emerged in the influent. The number of these newly emerged features almost doubled on days 7 and 8, accompanied by an increase in their intensities compared to day 6. The source of this irregularity was further investigated and pointed to junction C within the industrial park.

Interestingly, the observed pattern and the significant change in the influent’s composition on the 7th and 8th day were also reflected in the effluent, however with a 1-day delay. Hence, days 8 and 9 of the effluent appeared to have a distinct content compared to the other days.

#### Removal efficiency of the investigated WWTP

**Fig. 8:**
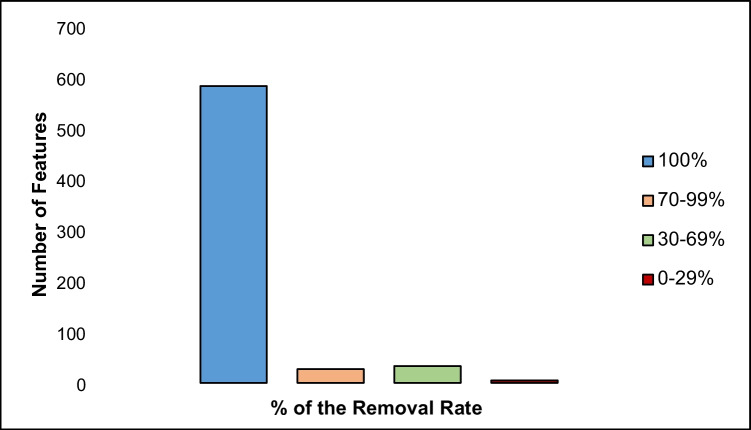
Distribution of removal rates for detected features in the WWTP. The removal rates are based on the intensities of the features detected in the influent and effluent. The bar graph illustrates the number of features corresponding to their respective removal rates

The data matrices of the influent and effluent were aligned, to create one feature list with their respective intensity profiles across both sampling sites. The removal efficiency of the WWTP was estimated by calculating the average intensities of the features in the influent and effluent over the 10-day period. The results are depicted in Fig. 8. Around 90% of the compounds were fully eliminated by the WWTP. The other substances were removed to a large extent and detected in the effluent at trace intensities. These results demonstrate the effective and strong performance of the WWTP, especially in treating industrial polar substances [8, 43, 44]. Just as in the case of all WWTPs, biological TPs were also detected in the effluent, which are a result of the treatment [45–47].

These findings are specific to this 10-day period. Highly sensitive targeted analytics, the state-of-the-art monitoring method today, is used continuously to monitor the efficiency of this WWTP.

#### Investigation of poorly removed features

**Fig. 9:**
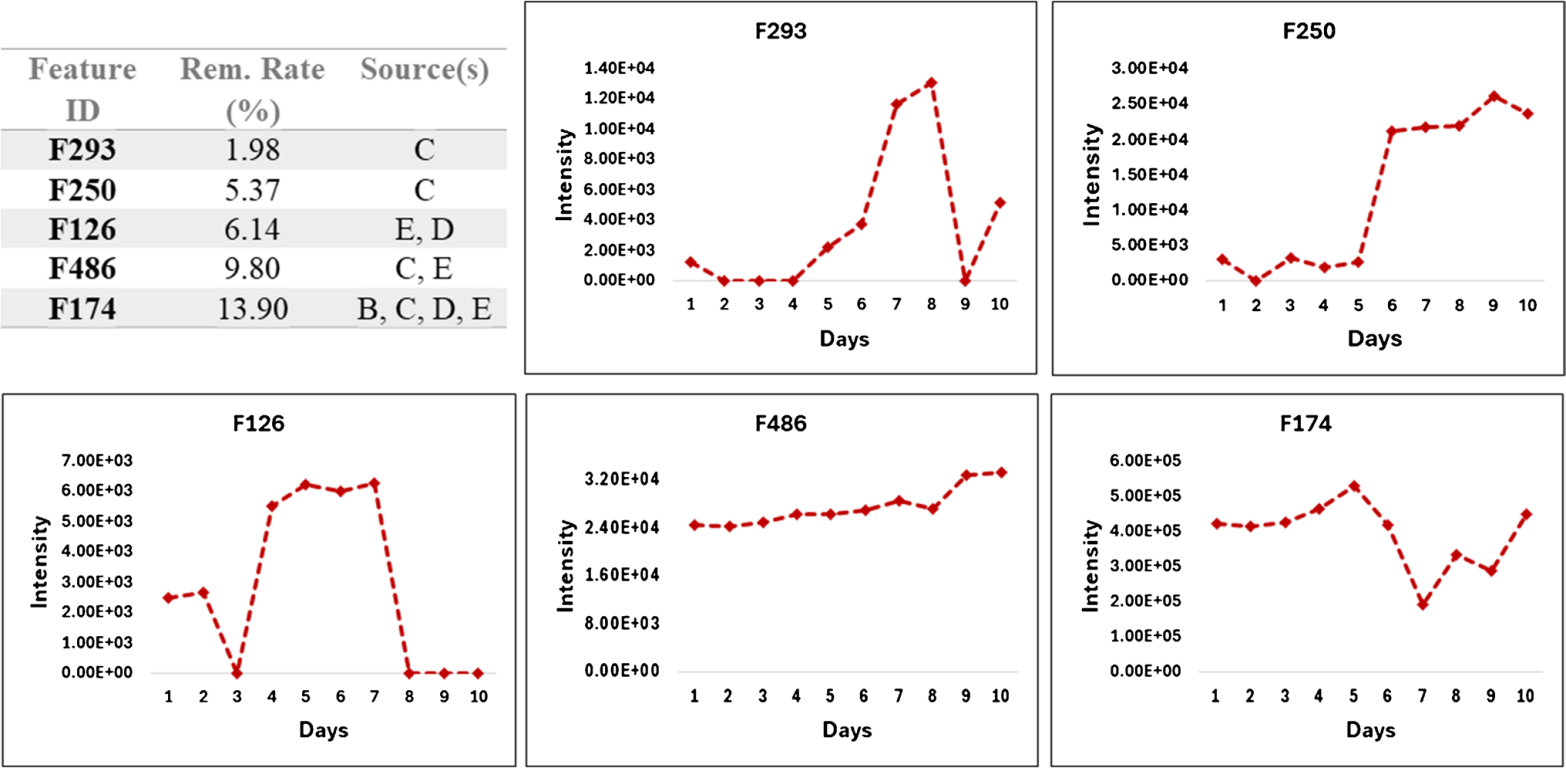
The features with the lowest removal rates and their intensity trends in the influent samples. These could be pinpointed to their emission source within the industrial park using the created chemical fingerprints (“Creation of a chemical fingerprint for the junctions” section)

Five of the substances with the lowest removal rate were prioritized and their intensity trends in the influent samples over the 10-day investigation period were plotted (Fig. 9). Feature F293 had the lowest removal rate of only 2%. The intensity plot of F293 indicates higher concentrations released in the wastewater between days 6 and 9. A plausible explanation for the low removal rate is that the WWTP microbiology may not be adapted to degrade this substance, especially when it is introduced in sudden high quantities. Similar observations were made for features F250 and F126, which had slightly higher removal rates of 5% and 6%, respectively. In the case of these features, extending the study duration could provide further insights into whether their poor removal is due to sudden high concentrations or if the WWTP microbiology is inherently unsuitable for their degradation. The degradation rate is specific to the physicochemical properties of the substance and the microbiology of the WWTP. Therefore, these substances may achieve higher removal rates over time as the WWTP adjusts to this change. Features F486 and F174 were present in high amounts in the influent throughout the investigation. Although these were partially removed, they can be considered as persistent polar compounds.

To take a step forward in reducing the amount of these substances in the wastewater, the sources of these compounds were investigated by employing junction chemical fingerprints. Two of the features, namely F293 and F250, could be exclusively traced back to junction C. F126 was detected at higher intensities (+E06) in samples from site D. It was also present in junction E due to the merging of wastewater from D into site E. Although F486 was detected in C and E, it is most probably released from one of the E plants, as the intensities in the E samples are higher by almost a factor of 10 in comparison to C. Interestingly, F174 was detected in all junctions with the exception of A. The next step involves identifying and notifying the specific chemical plants responsible for these emissions.

## Conclusion

Persistent polar compounds, highly soluble in water, pose challenges in water management. Traditional treatment methods struggle to remove them, and may even lead to the release of highly polar degradation products. This plays a role in their accumulation in our aquatic system and even our drinking water. In addition to their treatment, their analysis is also complex.

Herein, we propose a promising method to address these analytical challenges. The ZIC-HILIC stationary phase was coupled to HRMS, to perform NTS of highly polar compounds in industrial wastewater. The method provides the effective separation of such compounds, while employing NTS’ ability to monitor a sample’s chemical space without any prior knowledge about its composition. Optimized for industrial wastewater analysis, alongside RPLC-NTS, it enables the monitoring of a wider range of substances reaching a WWTP and discharging from it. This robust method captures contamination events or significant changes in the composition of influent and effluent, making it crucial for reducing the emissions of highly polar compounds.

Building on these results, the future work will focus on utilizing this method continuously aiming to prioritize and identify substances in the effluent. The application of this method is not limited to industrial wastewater. However, its performance for other matrices needs to be firstly examined.

## Supplementary Information

Below is the link to the electronic supplementary material.

Supplementary file1 (DOCX 2828 KB)
