# Supplementary material for: Enhanced industrial wastewater monitoring: method development for non-target screening of highly polar substances using ZIC-HILIC-HRMS
Source: Anal Bioanal Chem. 2024 Nov 16;417(1):167–81. doi: 10.1007/s00216-024-05635-9 (PMC11695456; doi:10.1007/s00216-024-05635-9)
Supplement: Supplementary file 1 — Supplementary file1 (DOCX 2828 KB) [file 216_2024_5635_MOESM1_ESM.docx]

Electronic supplementary material for:

**Enhanced Industrial Wastewater Monitoring: Method Development for Non-Target Screening of Highly Polar Substances Using ZIC-HILIC-HRMS**

## **Reyhaneh Armin^a,b^ , Jan Wachendorf^c^, Markus Weber^d^, Torsten C. Schmidt^a*^**

^a^ Faculty of Chemistry, Instrumental Analytical Chemistry, University of Duisburg- Essen, Universitätsstraße 5, 45141, Essen, Germany

^b^ Environmental Analysis, Currenta GmbH & Co. OHG, D-51368 Leverkusen, Germany

^c^Chemical pharmaceutical analysis - chromatography-mass spectrometry, Currenta GmbH & Co. OHG, D-51368 Leverkusen, Germany

^d^ Environmental Analysis, Currenta GmbH & Co. OHG, 41538 Dormagen, Germany

**^*^** Corresponding author:

Torsten C. Schmidt, e-mail: torsten.schmidt@uni-due.de, address: Faculty of Chemistry, Instrumental Analytical Chemistry, University of Duisburg-Essen, Universitätsstraße 5, 45141 Essen, Germany

**Table of contents**

[Table S1: List of substances used for method optimization, and QC samples. In the case of Chlormequat chloride, the [M+] of Chlormequat is listed. 4](#_Toc178678634)

[Table S2: Overview of the parameters tested in the 18 methods 11](#_Toc178678635)

[Table S3 HRMS acquisition parameters. These were used also during ZIC-HILIC acquisition 17](#_Toc178678636)

[Table S4: List of internal standards used in the C18 method 18](#_Toc178678637)

[Table S5: Parameters used in MZmine3 for feature extraction 19](#_Toc178678638)

[Figure S1: Log D values of the reference standards ranging from -7 to (highly polar) to 2 (non-polar) 5](#_Toc178869337)

[Figure S2: TIC of a measurement on mixed mode column a) Overall chromatogram, b) mass spectrum (min 3 to min 22) 6](#_Toc178869338)

[Figure S3: XICs of selected reference substances measured at pH values of 4.5, 6.0, 6.8, and 7.5. The pH value 6.8 was selected as it offered the best compromise of retention, FWHM, and Intensity 7](#_Toc178869339)

[Figure S4: XICs of selected reference substances measured at buffer concentrations of 5, 10 and 20 mM. The buffer concentration of 20mM was selected as it offered the best compromise of retention, FWHM, and Intensity 8](#_Toc178869340)

[Figure S5: The gradient systems tested throughout method optimization. Overall 9 gradient systems were investigated 10](#_Toc178869341)

[Figure S6: Scheme describing the weighting of the target and NTS key figures used for the ranking of the final 3 methods for both modii. 13](#_Toc178869342)

[Figure S7: %RSD of A) retention times and B) intensities of selected substances in positive mode measured in one sequence (n=10) in positive mode to determine the repeatability of the method in three different matrices. 14](#_Toc178869343)

[Figure S8: the % RSD of the intensities of selected standard compounds to determine the reproducibility of the method over a period of 10 days. 15](#_Toc178869344)

[Figure S9: The distribution of mass error of the internal standards added to all wastewater (influent and effluent) samples 20](#_Toc178869345)

[Figure S10: The distribution of the intensities of the internal standards added to all wastewater (influent and effluent) samples 21](#_Toc178869346)

[Figure S11: The distribution of the asymmetry of the internal standards added to all wastewater (influent and effluent) samples 21](#_Toc178869347)

[Figure S12: The distribution of the full width half maximum (FWHM) of the internal standards added to all wastewater (influent and effluent) samples 22](#_Toc178869348)

[Figure S13: The distribution of retention time shiffts of the internal standards added to all wastewater (influent and effluent) samples 22](#_Toc178869349)

# Method development and optimization

Table S1: List of substances used for method optimization, and QC samples. In the case of Chlormequat chloride, the [M+] of Chlormequat is listed.

| **Compound** | **CAS** | **Formula** | **[M+H]+** | **[M-H]-** | **Type** |
| --- | --- | --- | --- | --- | --- |
| **1,3-di-o-tolylguanidine** | 97-39-2 | C15H17N3 | 240.1495 | 238.135 | Basic |
| **3-Amino-1,2,4-triazole** | 61-82-5 | C2H4N4 | 85.0509 | 83.03632 | Basic |
| **4-Methoxy-6-(trifluoromethyl)-1,3,5-triazin-2-amine (MTTA)** | 5-7-5311 | C5H5F3N4O | 195.0488 | 193.0343 | Neutral |
| **5-Fluorouracile** | 51-21-8 | C4H3FN2O2 | 131.0251 | 129.0106 | Neutral |
| **Acephate** | 30560-19-7 | C4H10NO3PS | 184.0192 | 182.0046 | Neutral |
| **Acesulfam-K** | 55589-62-3 | C4H5NO4S | 164.0012 | 161.9867 | Acidic |
| **Aspartame** | 22839-47-0 | C14H18N2O5 | 295.1289 | 293.1143 | Amphoteric |
| **Cardiol (Pentetrazole)** | 54-95-5 | C6H10N4 | 139.0978 | 137.0833 | Acidic |
| **Chlormequat chloride** | 999-81-5 | C5H13ClN | 122.0730 [M^+^] | - | Cationic |
| **Cyanuric Acid** | 108-80-5 | C3H3N3O3 | 130.0247 | 128.0102 | Neutral |
| **Gabapentin** | 60142-96-3 | C9H17NO2 | 172.1332 | 170.1186 | Amphoteric |
| **Glyphosate** | 1071-83-6 | C3H8NO5P | 170.0213 | 168.006732 | Amphoteric |
| **Melamine** | 108-78-1 | C3H6N6 | 127.0727 | 125.0581 | Basic |
| **Metformin** | 657-24-9 | C4H11N5 | 130.1087 | 128.0942 | Basic |
| **Paracetamol** | 103-90-2 | C8H9NO2 | 152.0706 | 150.056 | Acidic |
| **Perfluorooctanoic acid** | 335-67-1 | C8HF15O2 | 414.9810 | 412.9664 | Acidic |
| **Picryl sulfonic acid** | 2508-19-2 | C6H3N3O9S | 293.9663 | 291.9517 | Acidic |
| **Saccharin** | 81-07-2 | C7H5NO3S | 184.0063 | 181.9917 | Acidic |
| **Thiourea** | 62-56-6 | CH4N2S | 77.0168 | 75.00224 | Neutral |
| **ε-Caprolactam** | 105-60-2 | C6H11NO | 114.0913 | 112.0768 | Neutral |

Figure S1: Log D values of the reference standards ranging from -7 to (highly polar) to 2 (non-polar)


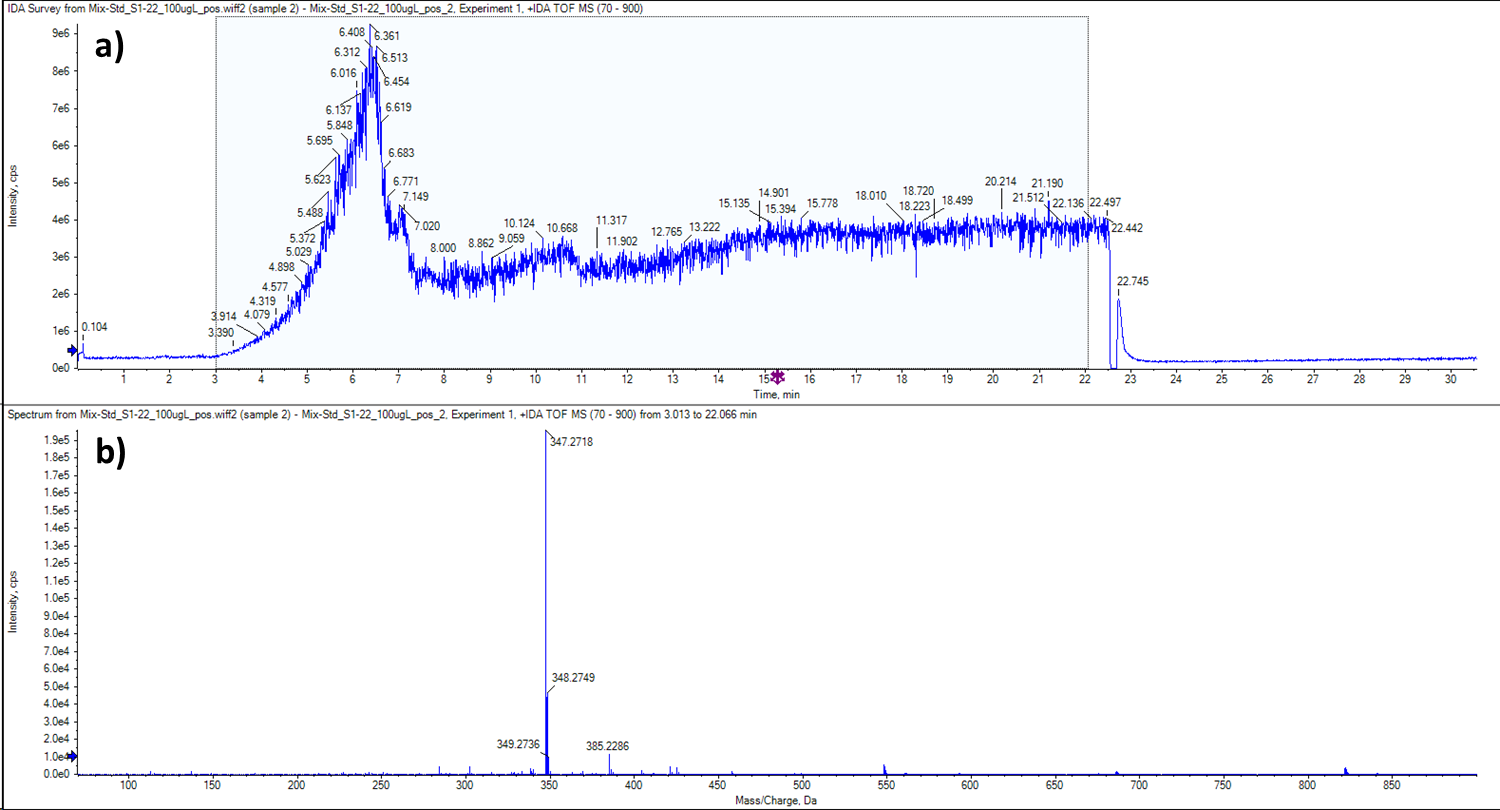


**B**

**A**

Figure S2: TIC of a measurement on mixed mode column a) Overall chromatogram, b) mass spectrum (min 3 to min 22)

Figure S3: XICs of selected reference substances measured at pH values of 4.5, 6.0, 6.8, and 7.5. The pH value 6.8 was selected as it offered the best compromise of retention, FWHM, and Intensity


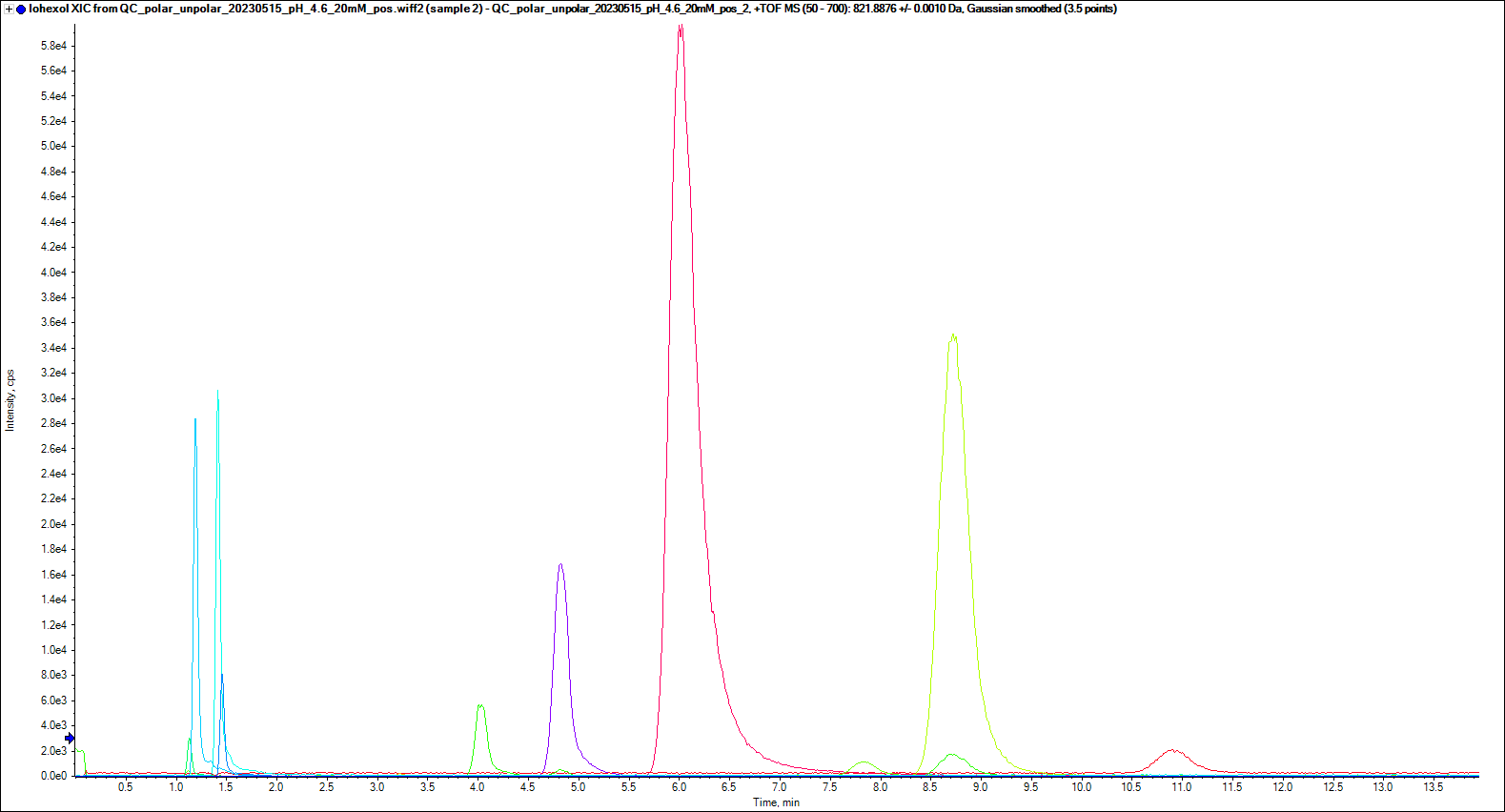

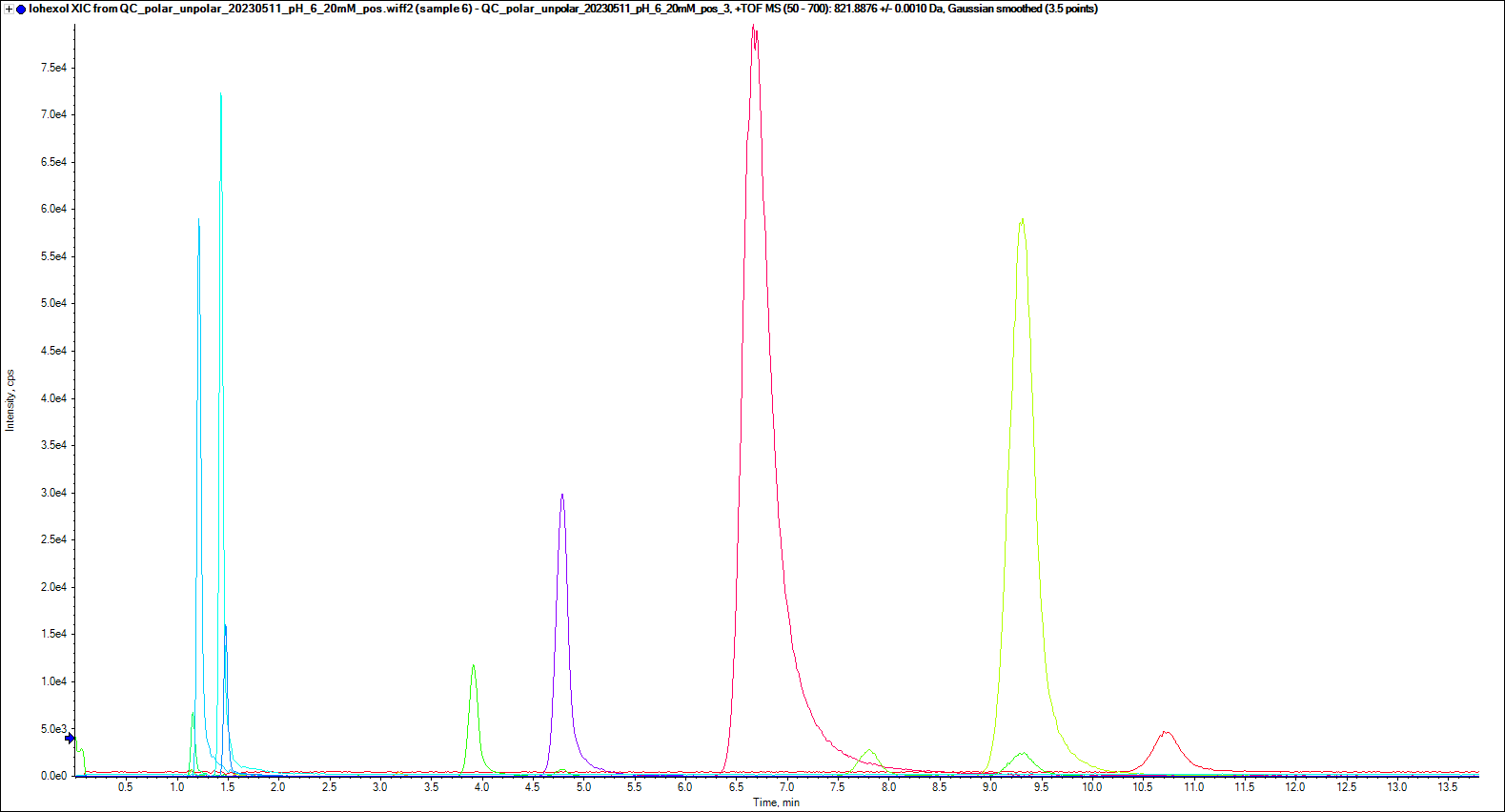


pH 7,5

pH 6,8

pH 6.0

pH 4.5


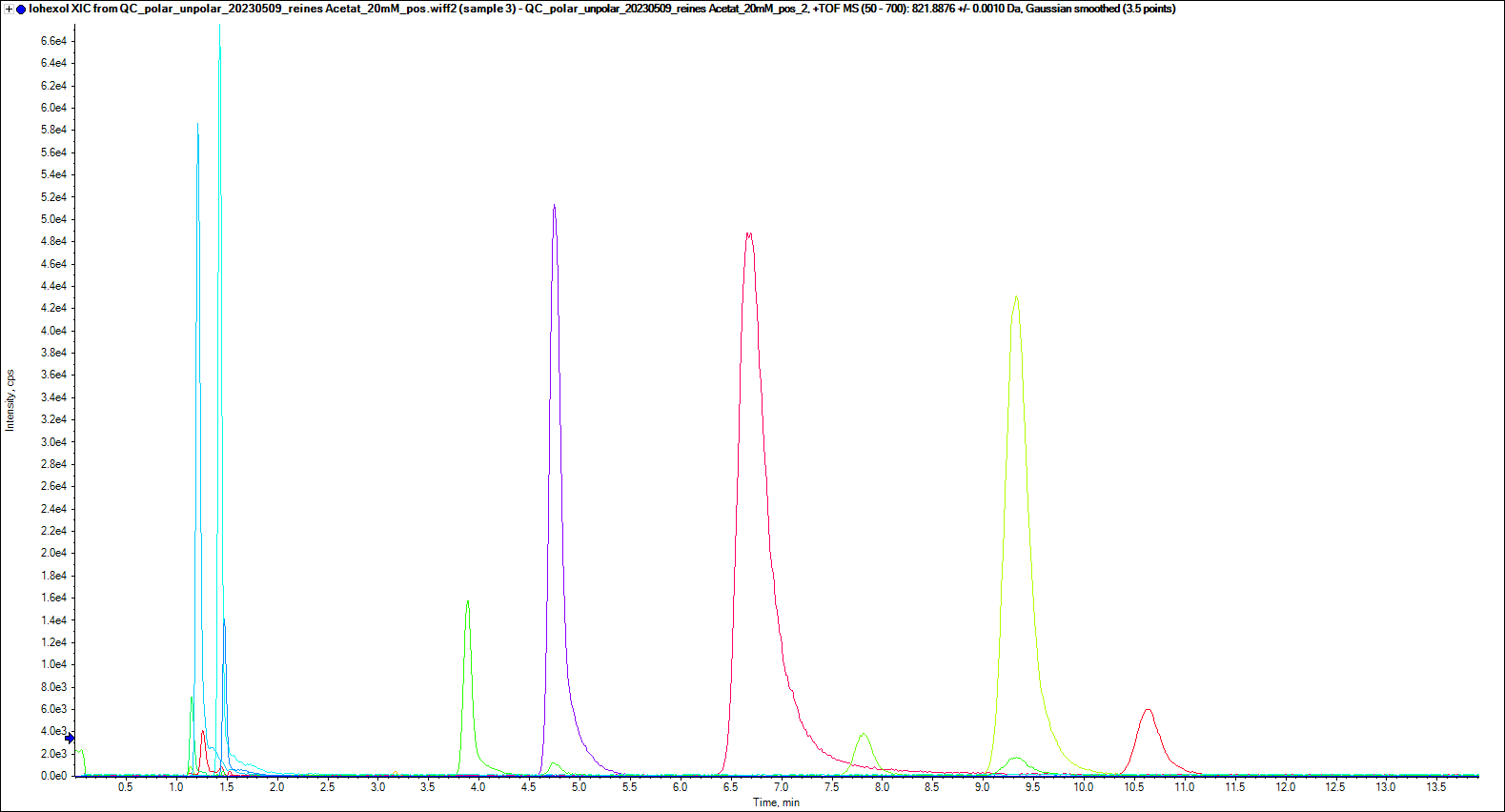

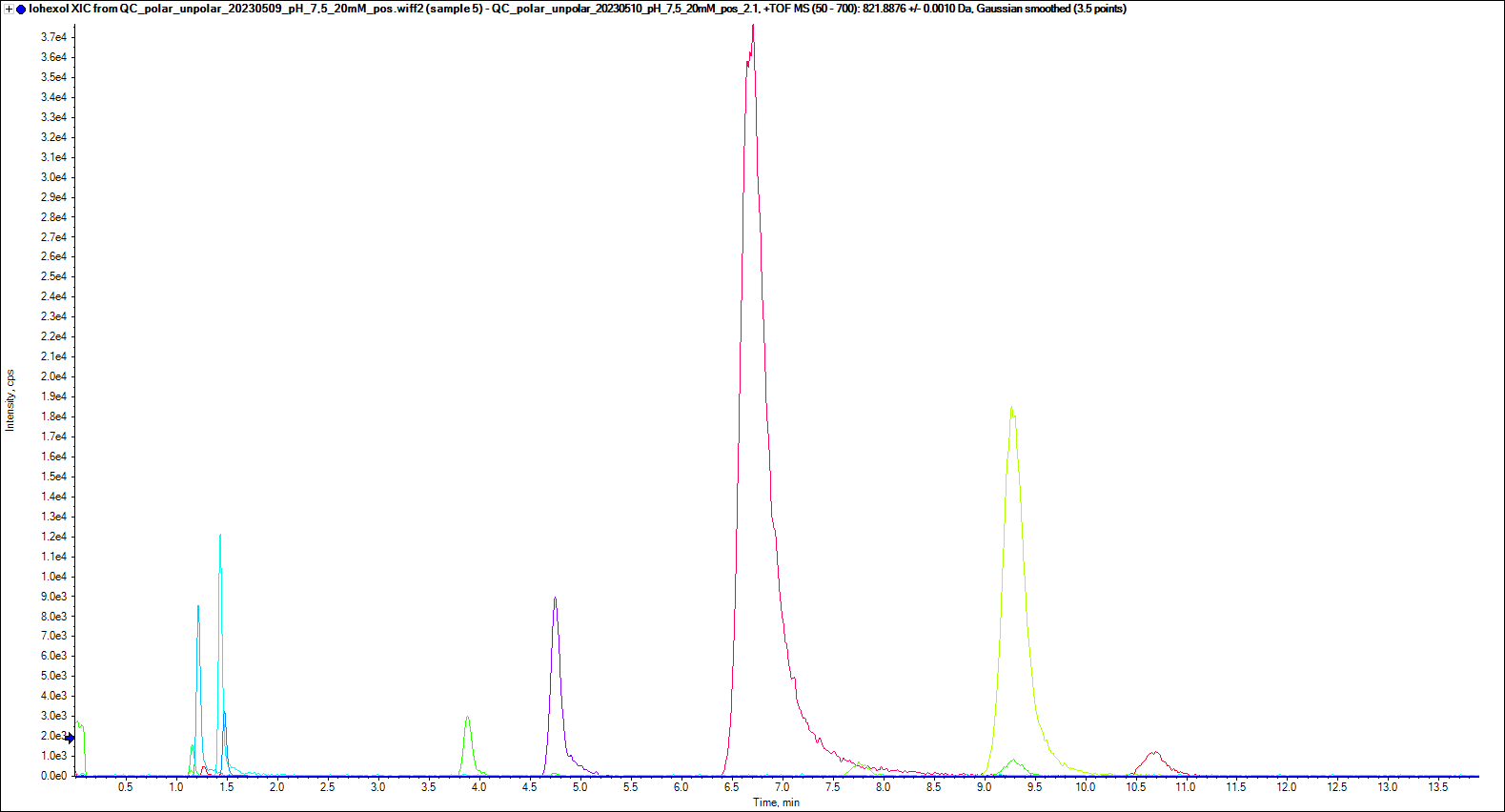


pH 7.5

pH 6.8

Melamin

Chlormequat

Gabapentin

Metformin

Aspartam

3-Amino-1,2,4-triazol

Caprolactam

Cardiol

Paracetamol


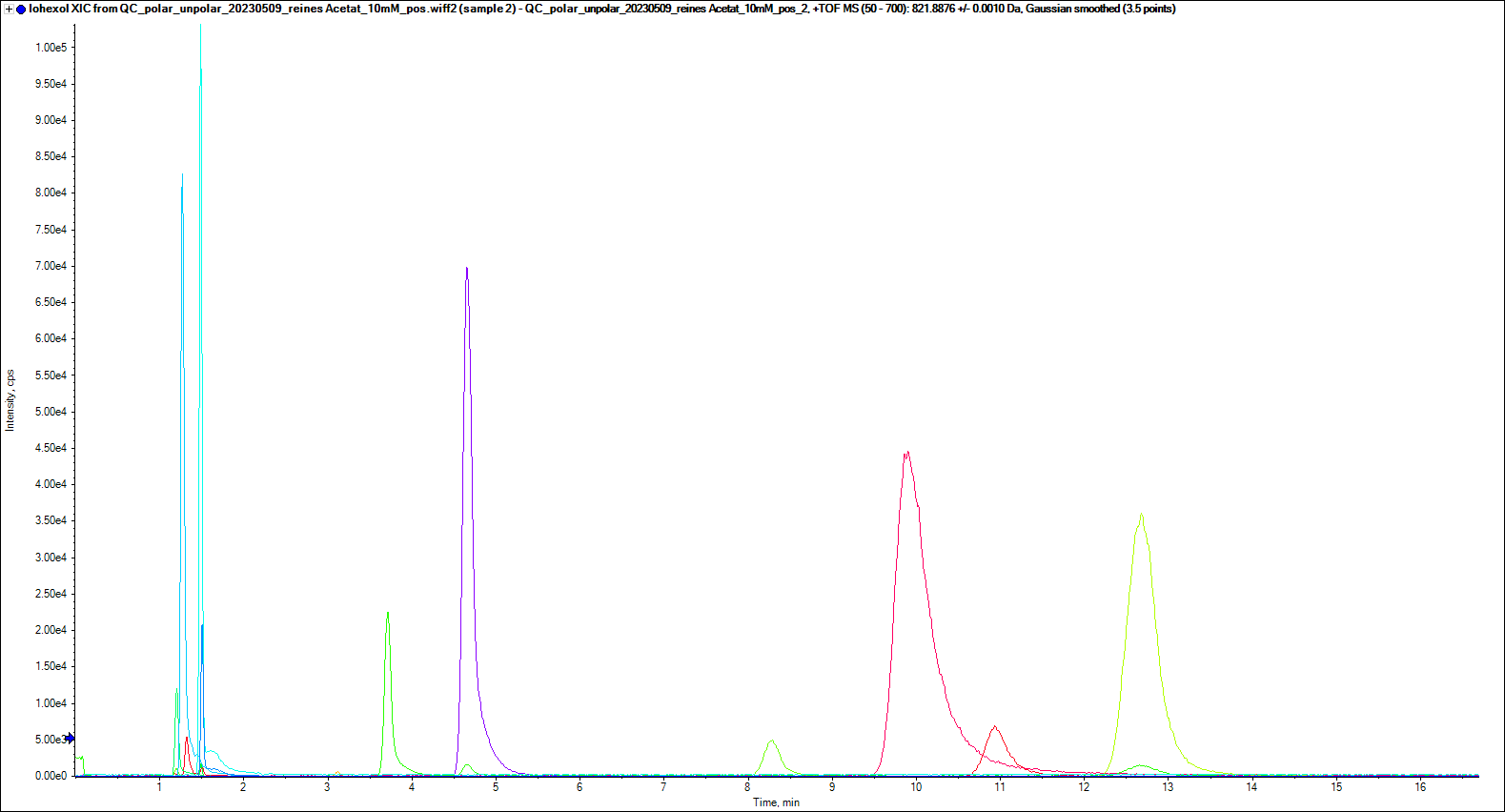

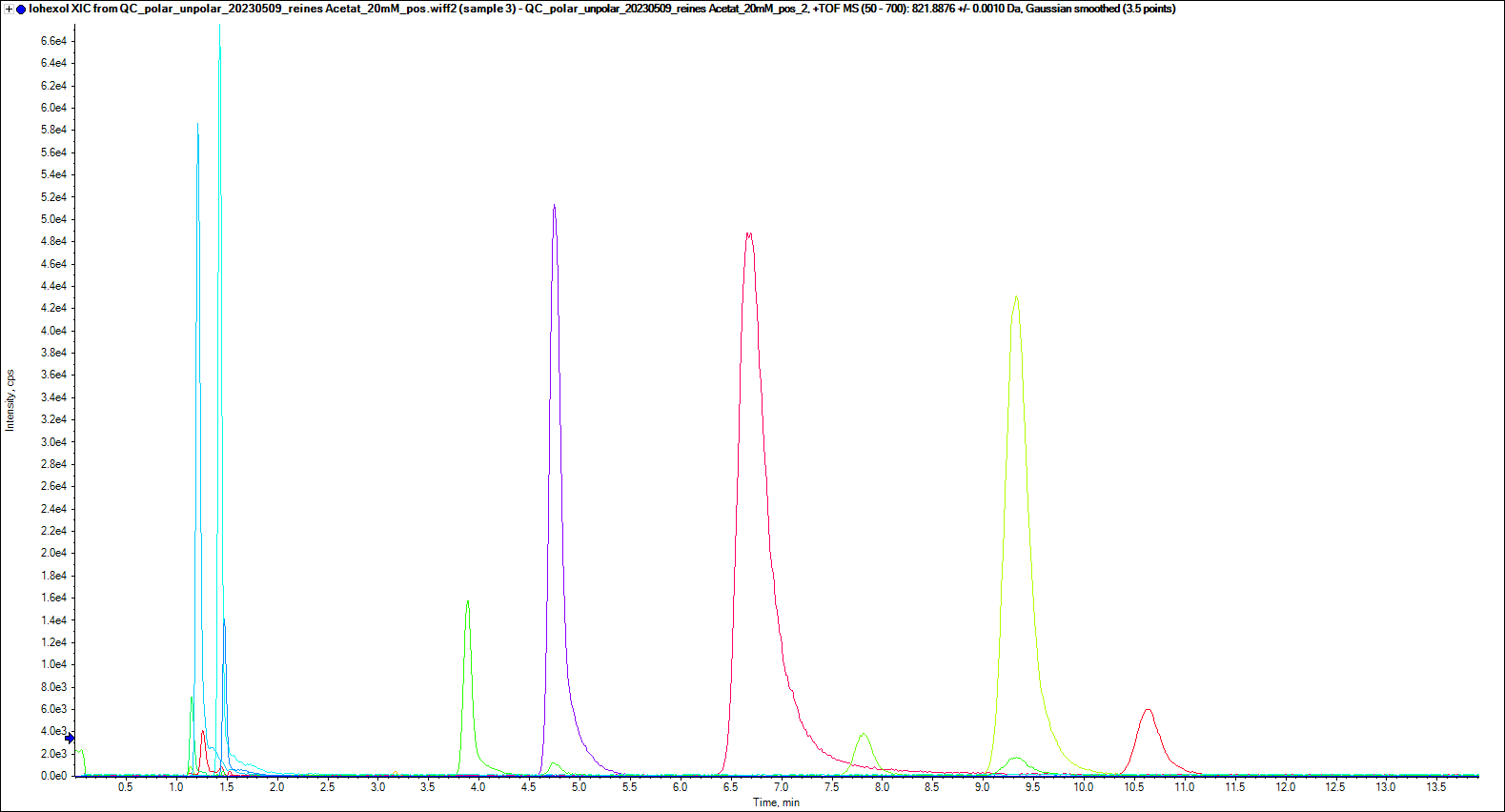


Gabapentin

Gabapentin

Caprolactam

Figure S4: XICs of selected reference substances measured at buffer concentrations of 5, 10 and 20 mM. The buffer concentration of 20mM was selected as it offered the best compromise of retention, FWHM, and Intensity


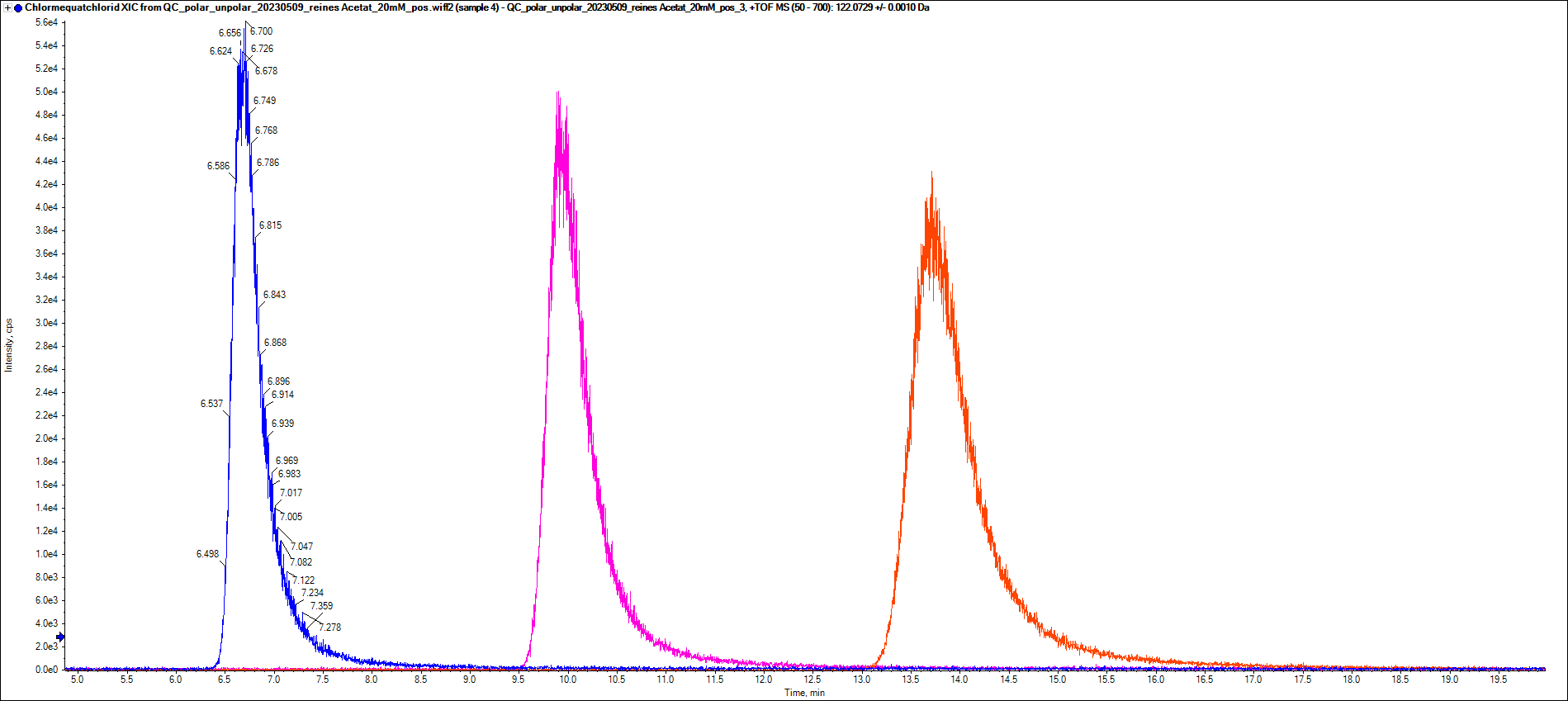


Chlormequat

20 mM

10 mM

5 mM


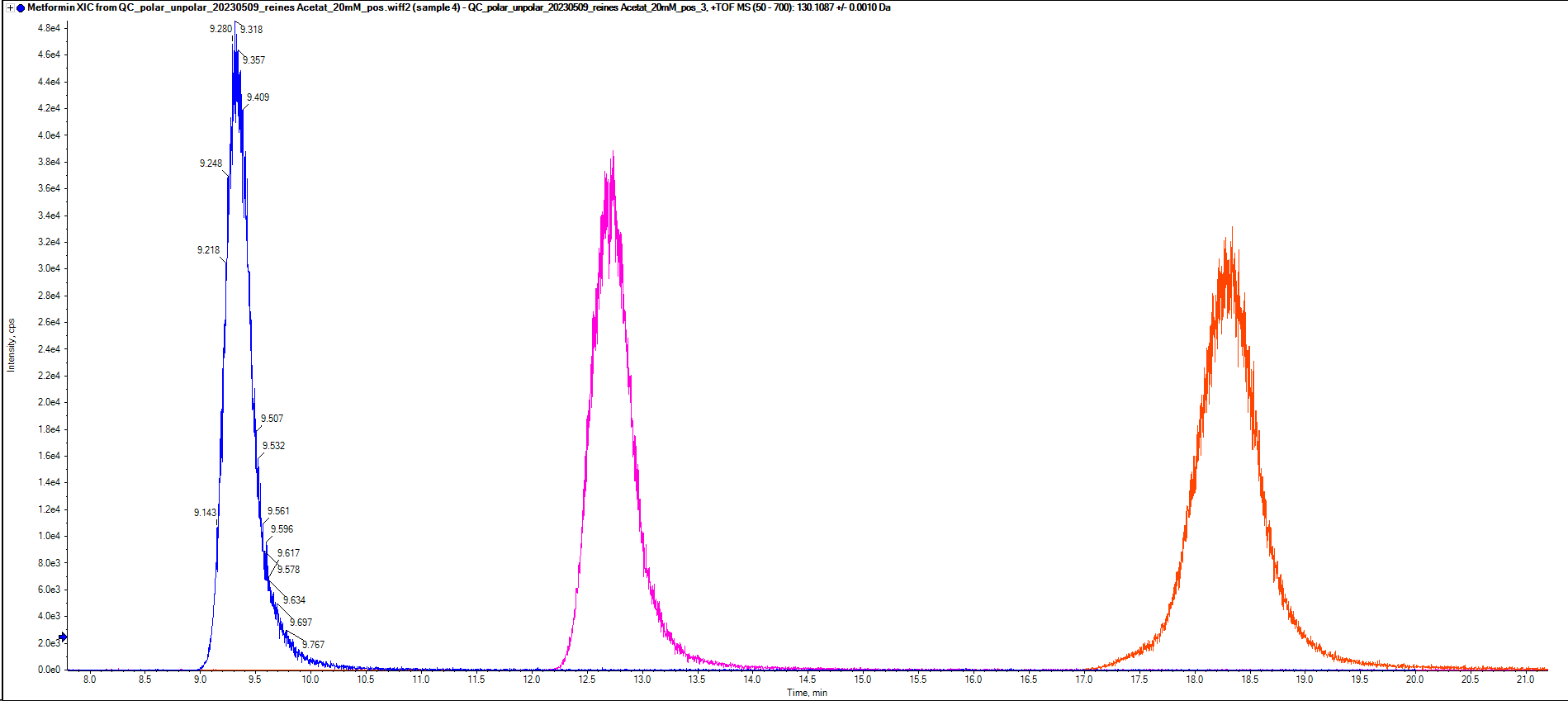


Metformin

20 mM

10 mM

5 mM


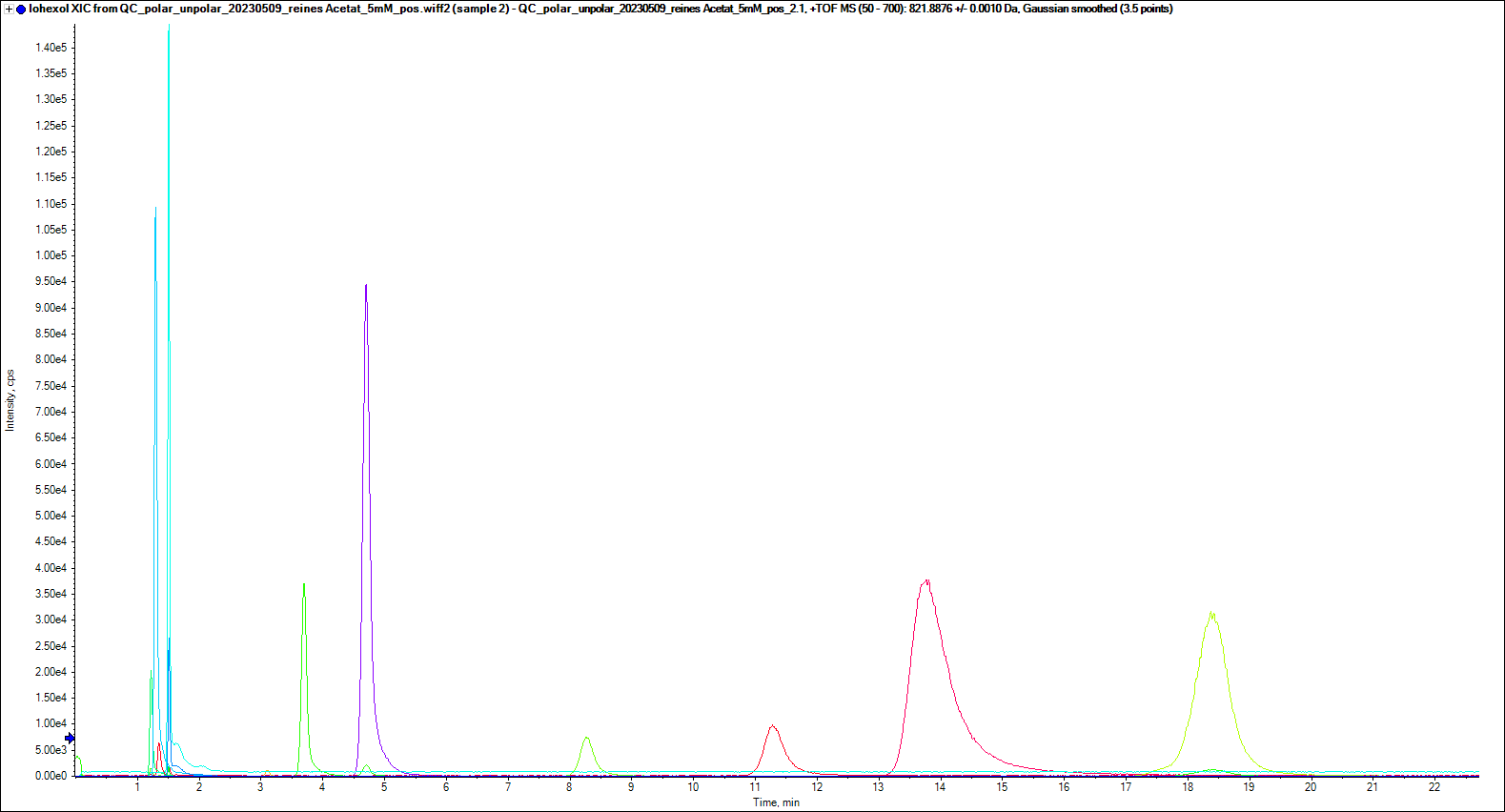


5 mM

Chlormequat

Metformin

Caprolactam

Cardiol

10 mM

20 mM

Chlormequat

Chlormequat

Melamin

Caprolactam

Paracetamol

Aspartam

Metformin

3-Amino-1,2,4-triazol

Cardiol

Metformin

Cardiol


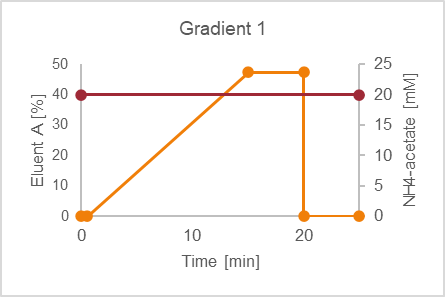

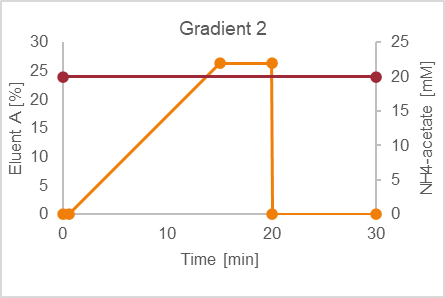

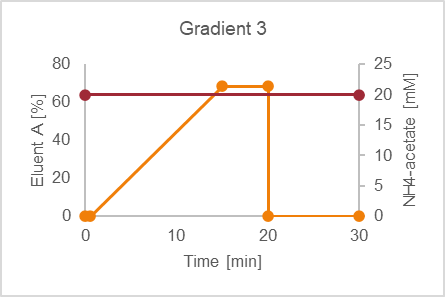

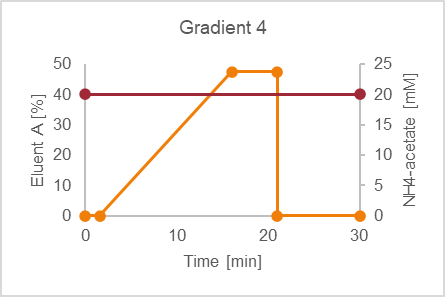

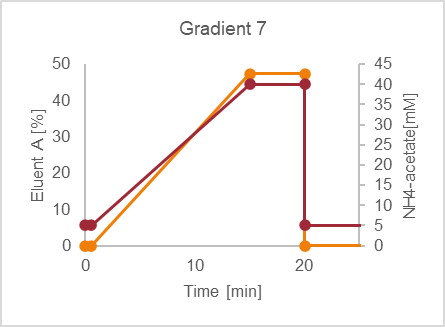

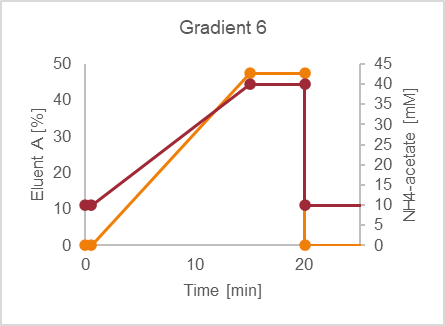

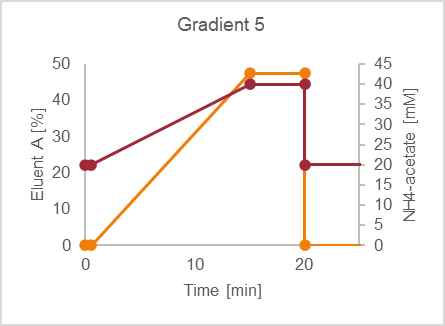

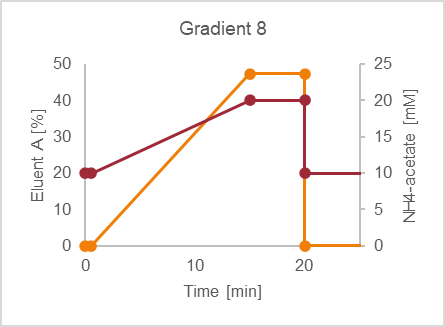


% Eluent A

NH4 Acetate concentration [mM]

Figure S5: The gradient systems tested throughout method optimization. Overall 9 gradient systems were investigated

Table S2: Overview of the parameters tested in the 18 methods

| **Method** | **1** | **2** | **3** | **4** | **5** | **6** | **7** | **8** | **9** |
| --- | --- | --- | --- | --- | --- | --- | --- | --- | --- |
| **Eluent A** | H_2_O+20mM NH_4_ Acetate | H_2_O+20mM NH_4_ Acetate | H_2_O+20mM NH_4_ Acetate | H_2_O+20mM NH_4_ Acetate | H_2_O+20mM NH_4_ Acetate | H_2_O+20mM NH_4_ Acetate | H_2_O+20mM NH_4_ Acetate | H_2_O+20mM NH_4_ Acetate | H_2_O+20mM NH_4_ Acetate |
| **Eluent B** | 95/5 ACN/H_2_O + 20mM NH_4_ Acetate | 95/5 ACN/H_2_O + 20mM NH_4_ Acetate | 95/5 ACN/H_2_O + 20mM NH_4_ Acetate | 95/5 ACN/H_2_O + 20mM NH_4_ Acetate | 95/5 ACN/H_2_O + 20mM NH_4_ Acetate | 95/5 ACN/H_2_O + 20mM NH_4_ Acetate | 95/5 ACN/H_2_O + 20mM NH_4_ Acetate | 95/5 ACN/H_2_O + 20mM NH_4_ Acetate | 95/5 ACN/H_2_O + 20mM NH_4_ Acetate |
| **pH** | 6.8 | 6.8 | 6.8 | 6.8 | 6.8 | 6.8 | 6.8 | 6.8 | 6.8 |
| **Gradient** | 1 | 1 | 1 | 1 | 1 | 2 | 3 | 4 | 1 |
| **Flow rate** | 0,50 | 0,40 | 0,30 | 0,50 | 0,50 | 0,50 | 0,50 | 0,50 | 0,30 |
| **Oven Temp.** | 35 | 35 | 35 | 50 | 65 | 35 | 35 | 35 | 65 |

Table S2 (continued): Overview of the parameters tested in the 18 methods

| **Method** | **10** | **11** | **12** | **13** | **14** | **15** | **16** | **17** | **18** |
| --- | --- | --- | --- | --- | --- | --- | --- | --- | --- |
| **Eluent A** | H_2_O+20mM NH_4_ Acetate | H_2_O+20mM NH_4_ Acetate | H_2_O+20mM NH_4_ Acetate | H_2_O+20mM NH_4_ Acetate | H_2_O+62.6mM NH_4_ Acetate | H_2_O+73.3mM NH_4_ Acetate | H_2_O+78.9mM NH_4_ Acetate | H_2_O+31.1mM NH_4_ Acetate | H_2_O+36.7mM NH_4_ Acetate |
| **Eluent B** | 95/5 ACN/H_2_O + 20mM NH_4_ Acetate | 95/5 ACN/H_2_O + 20mM NH_4_ Acetate | 95/5 ACN/H_2_O + 20mM NH_4_ Acetate | 95/5 ACN/H_2_O + 20mM NH_4_ Acetate | 95/5 ACN/H_2_O + 20mM NH_4_ Acetate | 95/5 ACN/H_2_O + 10mM NH_4_ Acetate | 95/5 ACN/H_2_O + 5mM NH_4_ Acetate | 95/5 ACN/H_2_O + 10mM NH_4_ Acetate | 95/5 ACN/H_2_O + 5mM NH_4_ Acetate |
| **pH** | 6.8 | 6.8 | 6.8 | 6.8 | 6.8 | 6.8 | 6.8 | 6.8 | 6.8 |
| **Gradient** | 1 | 1 | 1 | 1 | 5 | 6 | 7 | 8 | 9 |
| **Flow rate** | 0,35 | 0,35 | 0,30 | 0,40 | 0,40 | 0,40 | 0,40 | 0,40 | 0,40 |
| **Oven Temp.** | 35 | 45 | 45 | 45 | 35 | 35 | 35 | 35 | 35 |

# Scoring approach

Following the selection of the 3 most promising methods (section 2.9 of manuscript), the best of the three was selected using a second approach depicted in figure S7. In this approach, an even higher weighting was given to performance of the method in influent and effluent, in order to select the method most compatible with both matrices. Measurements were carried out in both modii.

The same target and NTS key figures were used and weighted as follows:


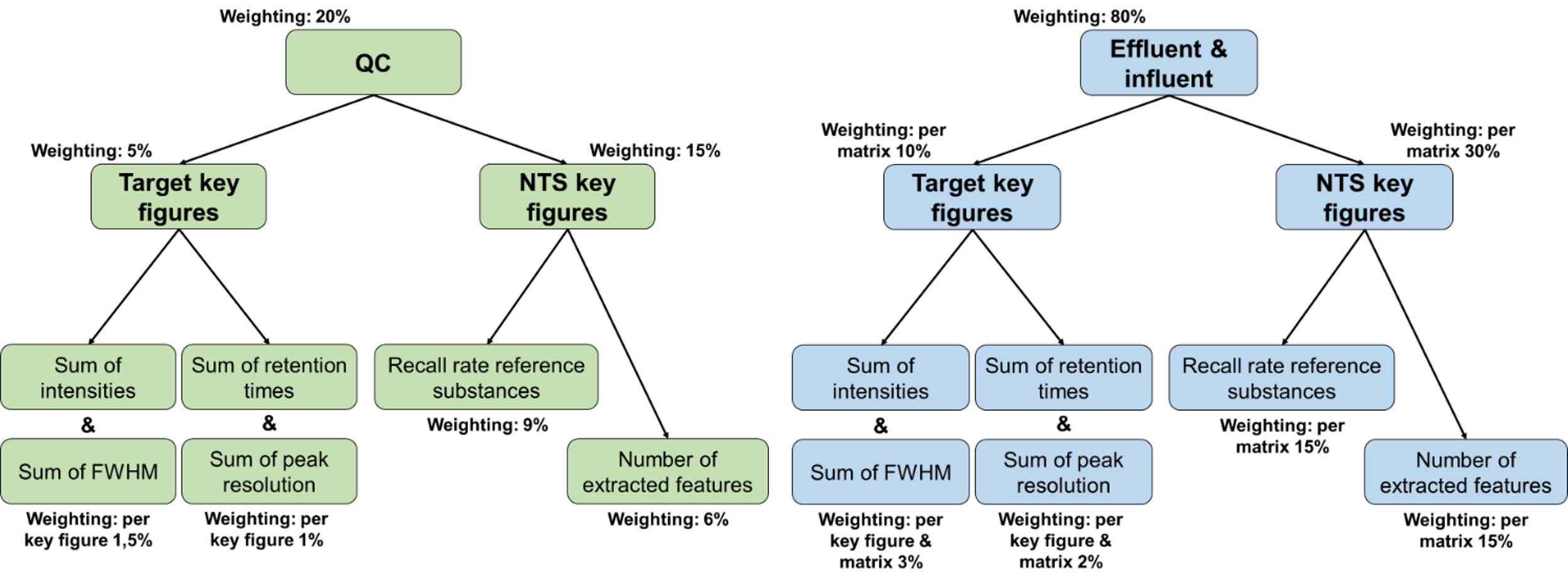


Figure S6: Scheme describing the weighting of the target and NTS key figures used for the ranking of the final 3 methods for both modii.

# Method Validation

## 3.1 Repeatability

**A**

**B**

Figure S7: %RSD of A) retention times and B) intensities of selected substances in positive mode measured in one sequence (n=10) in positive mode to determine the repeatability of the method in three different matrices.

## 3.2 Reproducibility

Figure S8: the % RSD of the intensities of selected standard compounds to determine the reproducibility of the method over a period of 10 days.

# Further Acquisition Parameters

Method of Boulard et al. for initial ZIC-HILIC method:

**Eluent A:** 10 mM ammonium formiate with 0.1% formic acid

**Eluent B**, 7.5 mM ammonium formiate in acetonitrile/Milli-Q, (90/10) with 0.1% formic acid.

Gradient system: 0🡪3 min: 100% B, 3🡪17 min: 100–75% B, 17🡪22 min: 75% B, 22🡪30 min 100% B.

Method of Montes et al. for initial mixed mode method:

**Eluent A:** Low organic content (2% acetonitrile and 5 mM ammonium acetate, pH 5.5)

**Eluent B:** High organic content (80% acetonitrile and 20 mM ammonium acetate, pH 5.5)

Transition from 100% eluent A to 100% eluent B within 10 minutes.

Method of Purschke et al. for C18 method:

**Eluent A**: Millipore Water with 0.1% formic acid

**Eluent B**: Methanol with 0.1% formic acid

Gradient system: 0.5🡪1.0 min: 10% B, 10🡪20 min: 90% B, 20🡪26 min: 90% B, 26🡪32 min: 0% B

Table S3 HRMS acquisition parameters. These were used also during ZIC-HILIC acquisition

| **HRMS Parameter** |
| --- |
| - HRMS system: SCIEX x500R qTOF - Ion source: Turbo V Electrospray ion source - Source temperature: 450 *°C* - Source voltage: 5500 V for positive and  -4500 V for negative - Source gas 1: 50 - Source gas 2: 70 - Curtain gas:40 - Measured mass range: 70-800 Da |

Table S4: List of internal standards used in the C18 method

| **Compound** | **CAS** | **Formula** | **[M+H]+** | **[M-H]-** |
| --- | --- | --- | --- | --- |
| Bezafibrate D6 | 1219802-74-0 | C19D6H14ClNO4 | 368.1526 | 366.1384 |
| Diuron D6 | 1007536-67-5 | C9D6H4Cl2N2O | 239.062 | 237.0473 |
| Metsulforon Methyl D3 | 2377723-88-9 | C14D3H12N5O6S | 385.1001 | 383.085 |

# Feature Extraction

Table S5: Parameters used in MZmine3 for feature extraction

| **Processing step** | **Parameter** |
| --- | --- |
| **Mass detection** | *Filter*   - Retention time: 0,7 - 20 min - MS level: 1   *Mass detector*  Centroid mit Noise level = 2000 |
| **ADAP Chromatogramm builder** | *Min group size in # of scans:* 10  *Group intensity threshold:* 1000  *Min highest intensity:* 2000   - *m/z tolerance:* 7 ppm |
| **Smoothing** | *Filter Width: 11* |
| **Chromatogramm Resolver** | *Algorithm: Wavelets (ADAP)*   - S/N threshold: 10 - S/N estimator: Intensity window SN - Min feature height: 2000 - Coefficient/area threshold: 110 - Peak duration range: 0 - 1 - RT wavelet range: 0 – 0,5   *m/z center calculation:* MEDIAN |
| **Isotope peak grouper** | *m/z tolerance:* 7 ppm  *Retention time tolerance:* 0,1 min  *Maximum charge:* 1  *Representative isotope:* Most intense |
| **Adduct search** | *RT tolerance:* 0,1 min   - [M+Na-H] 21,9825 m/z - [M+K-H] 37,9559 m/z - [M+NH3] 17,0265 m/z - Cl-Isotope: 1,9971 m/z - Br-Isotope: 1,9980   *m/z tolerance:* 7 ppm  *Max relative adduct peak height:* 80% |
| **Join aligner** | *m/z tolerance:* 7 ppm  *Weight for m/z:* 0,75  *RT tolerance:* 1 min  *Weight for RT:* 0,25 |

# Quality Assurance of the wastewater data

To ensure that the analytical system was performing properly, three of the reference substances were chosen and added to each wastewater sample as an internal standard. From the metadata it was known that these three substances are absent in the samples. These were also used to normalize the intensities and the RT of the features during the feature extraction process. The following box plots provide information on the distribution mass error, intensities, asymmetry factor, FWHM and retention time shifts of these standards.

The obtained results are satisfactory for our purpose. Interestingly, although the substance paracetamol showed higher %RSD compared to the other substances during the repeatability tests, (Fig 5 of the manuscript), during the wastewater investigation, it showed the lowest retention time shift. On the other hand, this compound showed a higher intensity distribution throughout the study, with the highest distribution observed in the influent samples. This may be because paracetamol is only moderately polar and is eluted in the first minutes of the measurement. As a result, it is highly influenced by the influent matrix, which consists of less polar compounds. Similar results were observed in the case of mass error.


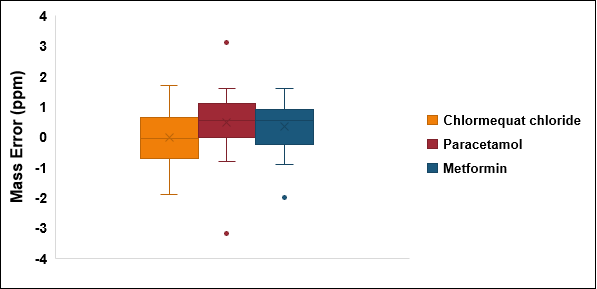


Figure S9: The distribution of mass error of the internal standards added to all wastewater (influent and effluent) samples


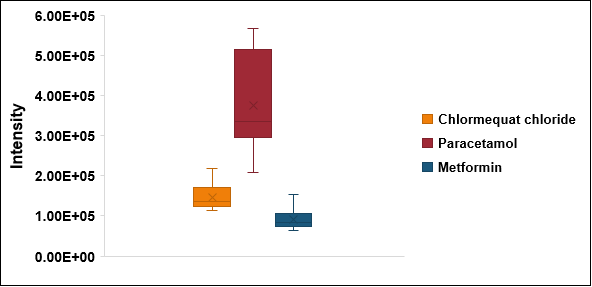


Figure S10: The distribution of the intensities of the internal standards added to all wastewater (influent and effluent) samples


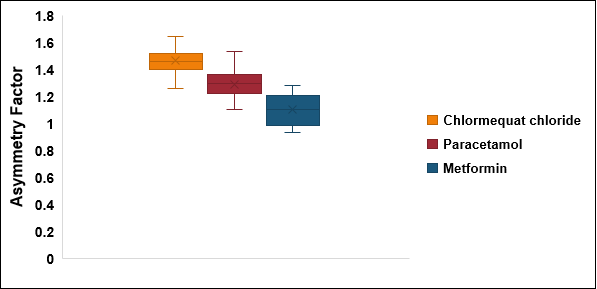


Figure S11: The distribution of the asymmetry of the internal standards added to all wastewater (influent and effluent) samples


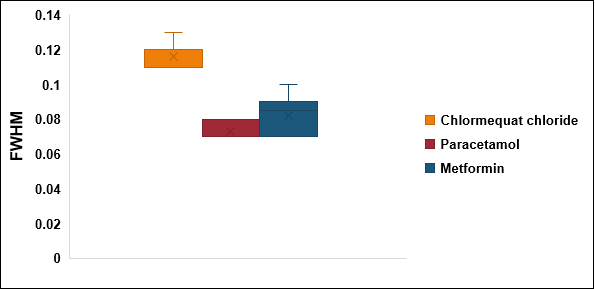


Figure S12: The distribution of the full width half maximum (FWHM) of the internal standards added to all wastewater (influent and effluent) samples


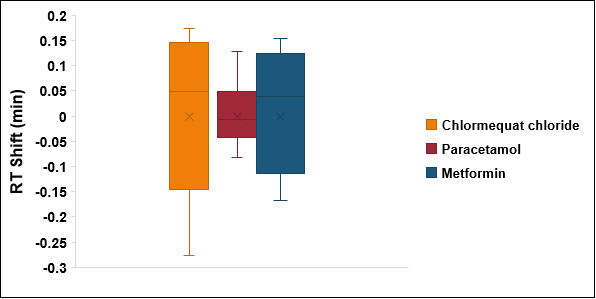


Figure S13: The distribution of retention time shiffts of the internal standards added to all wastewater (influent and effluent) samples
